# Exercise activates interferon response of the liver via Gpld1 to enhance antiviral innate immunity

**DOI:** 10.1126/sciadv.adk5011

**Published:** 2024-05-29

**Authors:** Tengfei Ren, Jiuyi He, Tingting Zhang, Anxing Niu, Yukang Yuan, Yibo Zuo, Ying Miao, Hongguang Zhang, Lichao Zang, Caixia Qiao, Xinhua Cao, Xinyu Yang, Zhijin Zheng, Yang Xu, Depei Wu, Hui Zheng

**Affiliations:** ^1^ Jiangsu Key Laboratory of Infection and Immunity, Institutes of Biology and Medical Sciences, Soochow University, 215123 Suzhou, Jiangsu, China.; ^2^ Department/Institute of Laboratory Medicine, Sichuan Provincial People's Hospital, School of Medicine, University of Electronic Science and Technology of China, Chengdu, Sichuan 611731, China.; ^3^ International Institute of Infection and Immunity, Institutes of Biology and Medical Sciences, MOE Key Laboratory of Geriatric Disease and Immunology of Ministry of Education of China, School of Medicine, Soochow University, Suzhou, Jiangsu 215123, China.; ^4^ Department of Infectious Diseases, The Affiliated Infectious Diseases Hospital, Suzhou Medical College of Soochow University, Suzhou, Jiangsu 215123, China.; ^5^ Department of Laboratory Medicine, The Third Affiliated Hospital of Soochow University, Changzhou 213003, China.; ^6^ Institute of Blood and Marrow Transplantation, Collaborative Innovation Center of Hematology, Soochow University, Suzhou, Jiangsu 215123, China.

## Abstract

Healthy behavioral patterns could modulate organ functions to enhance the body’s immunity. However, how exercise regulates antiviral innate immunity remains elusive. Here, we found that exercise promotes type I interferon (IFN-I) production in the liver and enhances IFN-I immune activity of the body. Despite the possibility that many exercise-induced factors could affect IFN-I production, we identified Gpld1 as a crucial molecule, and the liver as the major organ to promote IFN-I production after exercise. Exercise largely loses the efficiency to induce IFN-I in *Gpld1^−/−^* mice. Further studies demonstrated that exercise-produced 3-hydroxybutanoic acid (3-HB) critically induces Gpld1 expression in the liver. Gpld1 blocks the PP2A-IRF3 interaction, thus enhancing IRF3 activation and IFN-I production, and eventually improving the body’s antiviral ability. This study reveals that exercise improves antiviral innate immunity by linking the liver metabolism to systemic IFN-I activity and uncovers an unknown function of liver cells in innate immunity.

## Teaser

Exercise activates 3-hydroxybutanoic acid–Gpld1–IFN signaling axis in the liver that enhances antiviral immunity of the body.

## INTRODUCTION

The worldwide epidemic of severe acute respiratory syndrome coronavirus 2 and various unknown viruses has seriously affected the daily life of most people (*1*). Exercise, as a common behavioral pattern in daily life, is considered to be an effective way to boost the immune system. Proper exercise could reduce the risk of viral infections (*2*–*4*), suggesting that exercise may affect antiviral innate immunity of the body. However, the exact mechanisms have not been elucidated. Type I interferons (IFN-I and IFN-α/β) are important cytokines of antiviral innate immunity (*5*, *6*). IFN-I production is mediated by the activation of IFN regulatory factor 3 (IRF3) induced by the complex endogenous and exogenous stimuli (*7*–*9*). Subsequently, hundreds of IFN-stimulated genes (ISGs) are induced to fight pathogens, including viruses (*10*–*12*). Of note, IRF3 is a terminal signaling molecule of the well-known retinoic acid-inducible gene-I (RIG-I), cyclic GMP-AMP synthase-stimulator of interferon genes, and Toll-like receptor signaling pathways against viruses (*13*). Thus, exploring the delicate regulation of IRF3 activation and IFN-I response is critical for the understanding of the establishment of antiviral immune defense ability of the body.

Glycosyl-phosphatidylinositol (GPI)–specific phospholipase D1 (Gpld1) hydrolyzes inositol phosphate linkages in proteins anchored to the cell membrane, thus releasing these proteins from the membrane. Currently, little is known about the biological functions of Gpld1. Recent studies have found that Gpld1 promotes neurogenesis and improves cognition (*14*). In addition, prostasin secretion depends on GPI anchor cleavage by Gpld1 (*15*). Gpld1 detaches Cripto-1 from the cell membrane and may contribute to endothelial cell migration (*16*). Gpld1 has also been reported to be closely associated with diabetes (*17*, *18*). A report showed that Gpld1 is up-regulated in both the livers and the plasma of exercised mice (*19*). However, the mechanism by which Gpld1 is up-regulated after exercise and the biological significance of exercise-produced Gpld1 remains largely unknown.

Exercise enhances metabolism and could up-regulate a variety of metabolites. Recently, the regulation of the expression of key functional proteins by metabolites has attracted much attention. It has been reported that the metabolite 3-hydroxybutyric acid (3-HB) is produced primarily by the metabolism of fatty acids in the liver and can be up-regulated by starvation and ketogenic diets (*20*). The main role of 3-HB is to replace glucose in supplying energy to the body (*21*). However, there is growing evidence that 3-HB has a positive impact on the treatment of cardiovascular disease, cancer, nervous system diseases, and diabetes (*22*). So far, whether the metabolite 3-HB regulates immune response has been unreported. In this study, we revealed that exercise up-regulates Gpld1 expression by promoting 3-HB production in the liver. Gpld1 in turn inhibits the protein phosphatase 2A (PP2A)-mediated dephosphorylation of IRF3, leading to the activation of IRF3 and IFN-I production, which eventually enhances antiviral immunity of the body.

## RESULTS

### Exercise promotes IFN-I production that is positively correlated with Gpld1 expression in the liver

**Fig. 1.**
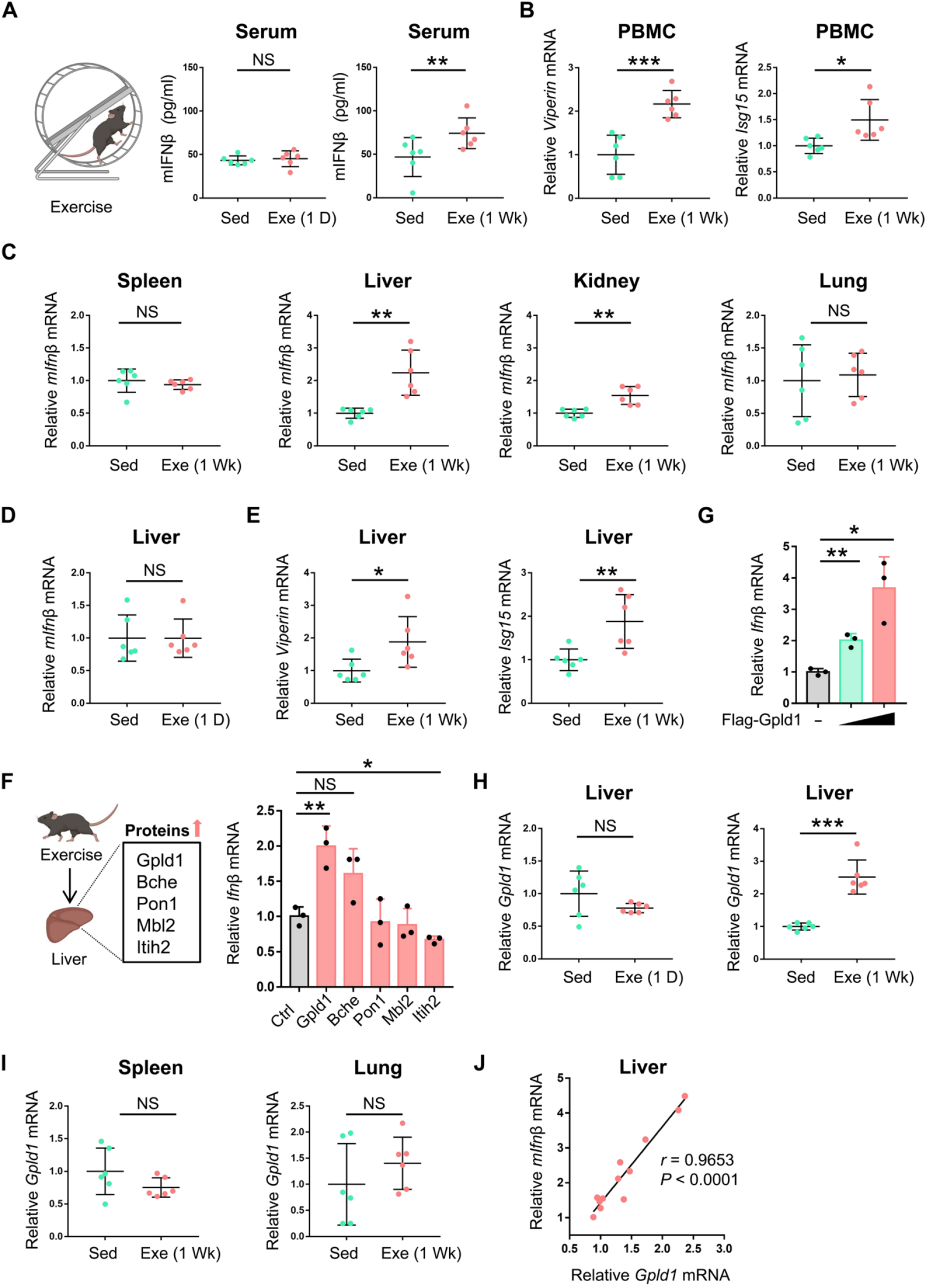
Exercise promotes IFN-I production that is positively correlated with Gpld1 expression in the liver. (**A**) Enzyme-linked immunosorbent assay (ELISA) analysis of mouse IFN-β (mIFN-β) protein levels in the serum of sedentary (Sed) mice (*n* = 6) and mice with 1 day (1 D) or 1 week (1 Wk) of exercise (Exe) (*n* = 6). This model in (A) was created with BioRender.com. (**B**) Real-time quantitative polymerase chain reaction (RT-qPCR) analysis of *Viperin* and *Isg15* mRNA levels in the peripheral blood mononuclear cells (PBMCs) from sedentary mice (*n* = 6) and exercised mice (1 Wk; *n* = 6). (**C**) RT-qPCR analysis of *mIfn*β mRNA levels in the spleens, livers, kidneys, and lungs from (B). (**D**) RT-qPCR analysis of *mIfn*β mRNA levels in the livers of sedentary mice (*n* = 6) and exercised mice (1 D; *n* = 6). (**E**) RT-qPCR analysis of *Viperin* and *Isg15* mRNA levels in the livers from (B). (**F**) Top five proteins up-regulated by exercise in mouse livers were analyzed and listed based on the reported study (*19*). RT-qPCR was used to analyze *Ifn*β mRNA levels in HepG2 cells transfected with control vectors (Ctrl) or the vector expressing each of these five proteins. This model in (F) was created with BioRender.com. (**G**) RT-qPCR analysis of *Ifn*β mRNA levels in HepG2 cells transfected with increasing amounts of Flag-Gpld1. (**H**) RT-qPCR analysis of *Gpld1* mRNA levels in the livers from (A). (**I**) RT-qPCR analysis of *Gpld1* mRNA levels in the spleens and lungs from (B). (**J**) Linear regression analysis of the correlation between *Gpld1* mRNA and *mIfn*β mRNA levels in the livers of exercised mice (1 Wk; *n* = 12). NS, not significant (*P* > 0.05). *P < 0.05, **P < 0.01, and ***P < 0.001 (two-tailed unpaired Student’s *t* test). Data are shown as the mean and SD of six [(A) to (E), (H), and (I)] or 12 (J) individual mice, or three [(F) and (G)] biological replicates.

To explore the potential regulation of antiviral innate immunity by exercise, we established an exercised mouse model according to the reported protocols (Fig. 1A) (*19*). We observed that 1 day of exercise did not significantly affect mouse serum IFN-I levels; however, a continuous week of exercise significantly resulted in an increase in serum IFN-I levels (Fig. 1A). Consistently, the expression levels of ISGs, including the representative ISGs *Viperin* and *Isg15*, were up-regulated in the peripheral blood mononuclear cells (PBMCs) of exercised mice compared with those of sedentary mice (Fig. 1B), suggesting an enhancement of IFN-I antiviral activity of the body. Next, we determined which organs are the major sources of increased serum IFN-I induced by exercise. To this end, several mouse organs, including the spleen, liver, kidney, and lung, were collected and then IFN-I expression was analyzed. We found that there was a significant increase in IFN-I expression in the liver, as well as the kidney, after 1 week of exercise, and the increase was much more pronounced in the liver, which is similar with the report showing that 67% of exercise-induced factors in mature mice are mainly expressed in the livers (*19*, *23*, *24*). In contrast, there was no significant increase in IFN-I levels in either mouse spleen or lung tissues after exercise (Fig. 1C). However, 1 day of exercise cannot up-regulate IFN-I levels in the livers of mice (Fig. 1D), which is consistent with the observation that 1 day of exercise did not change serum IFN-I levels (Fig. 1A). Further observation of up-regulated ISGs expression in the livers of mice with 1 week of exercise also demonstrated the increase in IFN-I levels in mouse livers (Fig. 1E).

Given that the liver is the organ showing the strongest induction of IFN-I production after 1 week of exercise, we next sought to determine how exercise promotes IFN-I production in the liver. Thus far, exercise-induced factors have actually been extensively observed in various studies, which provides very rich resources for exploring the mechanism of exercise-induced IFN-I production in the liver. Given that many of exercise-induced factors could be involved in IFN-I production to some extent, we analyzed a recent comprehensive study on exercise-induced proteins in the livers and the plasma of mice (*19*), aiming at focusing on those well-recognized exercise-induced factors to study IFN-I response in the liver after exercise. Thus, the top five up-regulated proteins in the livers after exercise, including Gpld1, Bche, Pon1, Mbl2, and Itih2, were first elected from this reported study for further analysis (Fig. 1F) (*19*). We found that overexpression of Gpld1 but not the other four proteins strongly promoted IFN-I production in the liver cells (Fig. 1F), and Gpld1 can up-regulate IFN-I expression in a dose-dependent manner (Fig. 1G). In line with the previous report (*19*), we found that 1 week of exercise significantly up-regulated Gpld1 mRNA levels in the liver (Fig. 1H, right), but 1 day of exercise did not (Fig. 1H, left). In addition, 1 day of exercise did not significantly increase Gpld1 levels in the spleens of mice (fig. S1A). Consistent with Gpld1 mRNA expression, Gpld1 protein levels were also up-regulated in the livers of mice with 1 week of exercise (fig. S1B). However, 1 week of exercise did not up-regulate Gpld1 expression in both the spleens and the lungs (Fig. 1I) but slightly up-regulated Gpld1 levels in the kidneys of mice (fig. S1C). We found a significant correlation between Gpld1 expression and IFN-I levels in the livers of exercised mice (Fig. 1J). Together, these findings suggested that exercise stimulates IFN-I production in the liver and enhances IFN-I activity in the peripheral blood, and exercise-elevated IFN-I levels are positively correlated with Gpld1 expression in the liver.

### Gpld1 enhances IFN-I antiviral immunity in response to viral infection

**Fig. 2.**
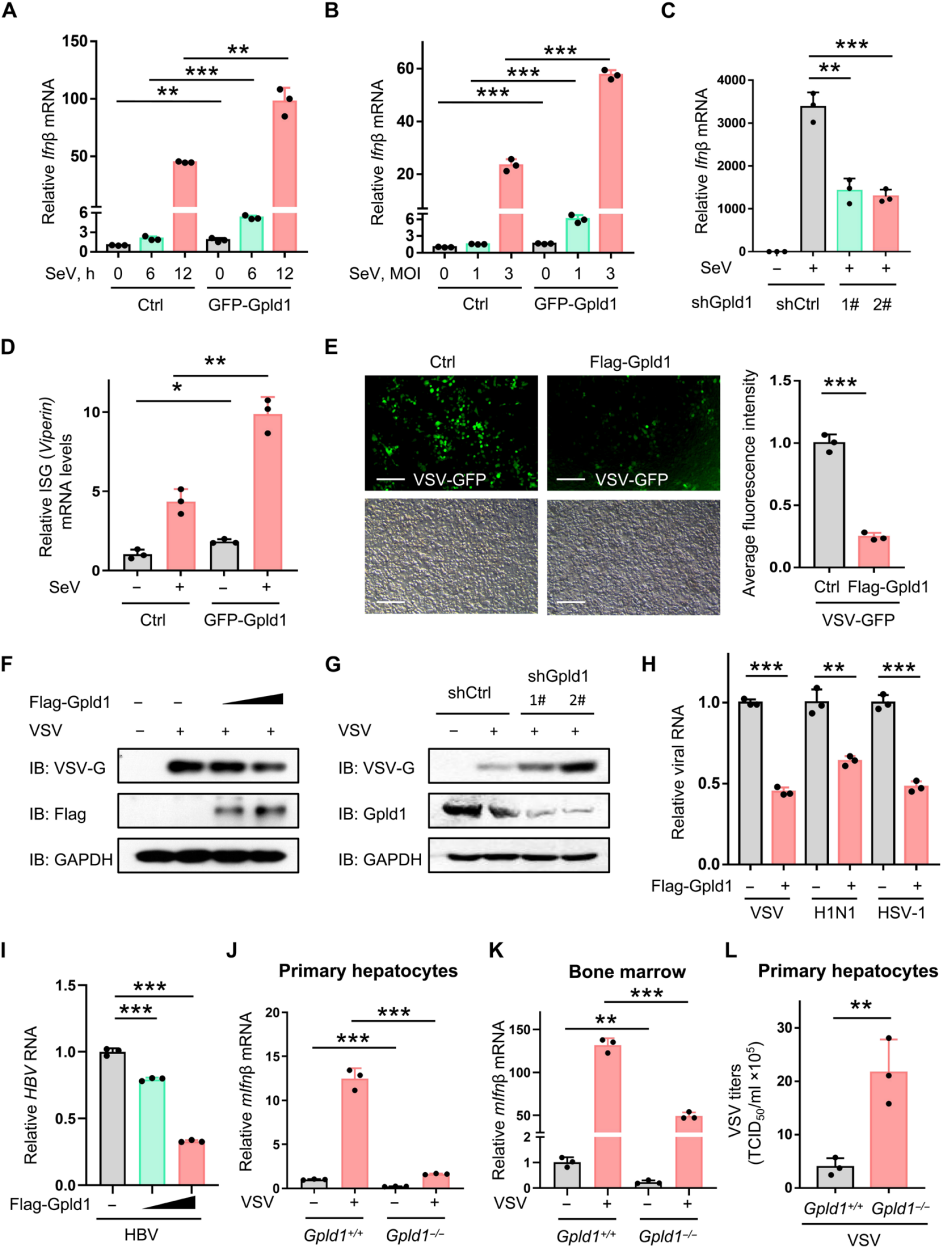
Gpld1 enhances IFN-I antiviral immunity in response to viral infection. (**A** to **C**) RT-qPCR analysis of *Ifn*β mRNA in HEK293T cells transfected with control vectors (Ctrl) or GFP-Gpld1, and then infected with Sendai virus (SeV) [multiplicity of infection (MOI) = 1.0] as indicated (A), or infected with SeV (MOI = 1.0 and 3.0; 12 hours) (B), or transfected with control shRNAs (shCtrl) or shGpld1 (1# or 2#) then infected with SeV (MOI = 1.0; 12 hours) (C). (**D**) RT-qPCR analysis of *Viperin* mRNA in HEK293T cells transfected with control vectors or GFP-Gpld1, and then infected with SeV as (C). (**E**) Fluorescence microscopy of the VSV virus with a GFP gene (VSV-GFP) in HepG2 cells transfected with control vectors or Flag-Gpld1, and then infected with VSV (MOI = 1.0) for 24 hours. Scale bars, 100 μm. (**F**) Western blot analysis of VSV-encoded G protein (VSV-G) in HepG2 cells transfected with increasing amounts of Flag-Gpld1, and then infected with VSV as (E). IB, immunoblot. (**G**) Western blot analysis of VSV-G in HepG2 cells transfected with shGpld1 (1# or 2#), and then infected with VSV-GFP (MOI = 1.0) for 24 hours. (**H**) RT-qPCR analysis of virus RNA levels in HEK293T cells transfected with Flag-Gpld1, and then infected with VSV/H1N1/HSV-1 (MOI = 1.0) for 24 hours. (**I**) RT-qPCR analysis of HBV RNA levels in HepG2 cells cotransfected with HBV-1.3 constructs and Flag-Gpld1. (**J** and **K**) RT-qPCR analysis of *mIfn*β mRNA levels in *Gpld1^+/+^* or *Gpld1^−/−^* mouse primary hepatocytes (J) or mouse bone marrow cells (K) infected with VSV (MOI = 1.0) for 12 hours. (**L**) Tissue culture-infective dose (TCID_50_) assay of virus titers in culture supernatants from (J). *P < 0.05, **P < 0.01, and ***P < 0.001 (two-tailed unpaired Student’s *t* test). Data are shown as mean and SD of three biological replicates [(A) to (D) and (H) to (L)], or are representative of three independent experiments [(F) and (G)].

Gpld1 is well recognized as a phospholipase that can detach GPI-anchored proteins. So far, whether Gpld1 is involved in antiviral innate immunity remains unknown. Thus, we further explored the potential role of Gpld1 in regulating IFN-I antiviral response. We noticed that Gpld1 overexpression not only up-regulated basal levels of IFN-I but also enhances virus-induced IFN-I production (Fig. 2, A and B). Conversely, knockdown of Gpld1 reduced IFN-I production during virus infection (Fig. 2C). Consistently, Gpld1 overexpression promoted virus-induced expression of ISGs (Fig. 2D and fig. S2A), while Gpld1 knockdown attenuated ISG expression during virus infection (fig. S2B). These results suggested that Gpld1 facilitates IFN-I production and IFN-I antiviral activity.

Next, we investigated whether Gpld1 could enhance cellular antiviral immunity. Using fluorescence microscopy, we observed that overexpression of Gpld1 inhibited cellular infection of a vesicular stomatitis virus (VSV) with a green fluorescent protein (GFP) gene (Fig. 2E), and Gpld1 overexpression inhibited VSV infection in a dose-dependent manner (Fig. 2F). In contrast, Gpld1 knockdown promoted virus infection (Fig. 2G). Gpld1 exerted broad-spectrum antiviral effects, as shown by the observations that Gpld1 inhibited infection of RNA viruses, including VSV and influenza A virus (H1N1), as well as a DNA virus, herpes simplex virus (HSV-1) (Fig. 2H and fig. S2, C and D), which is consistent with Gpld1-mediated up-regulation of IFN-I production and ISG expression during viral infection. In addition, overexpression of Gpld1 can reduce hepatitis B virus (HBV) RNA levels in HepG2 cells in a dose-dependent manner (Fig. 2I).

On the basis of these findings, we further used *Gpld1^+/+^* and *Gpld1^−/−^* mouse primary cells to analyze the intrinsic role of cellular Gpld1 in IFN-I production. We found that both basal and VSV-induced IFN-I levels in the primary hepatocytes or bone marrow cells derived from *Gpld1^−/−^* mice were significantly lower than those from *Gpld1^+/+^* cells (Fig. 2, J and K). Moreover, the primary hepatocytes from *Gpld1^−/−^* mice showed more severe virus infection, compared with those from *Gpld1^+/+^* mice (Fig. 2L). Collectively, these findings demonstrated that Gpld1 is a positive regulator of IFN-I production, and Gpld1 enhances IFN-I immune response when encountering virus infection.

### Gpld1 targets IRF3 and promotes IRF3 phosphorylation

**Fig. 3.**
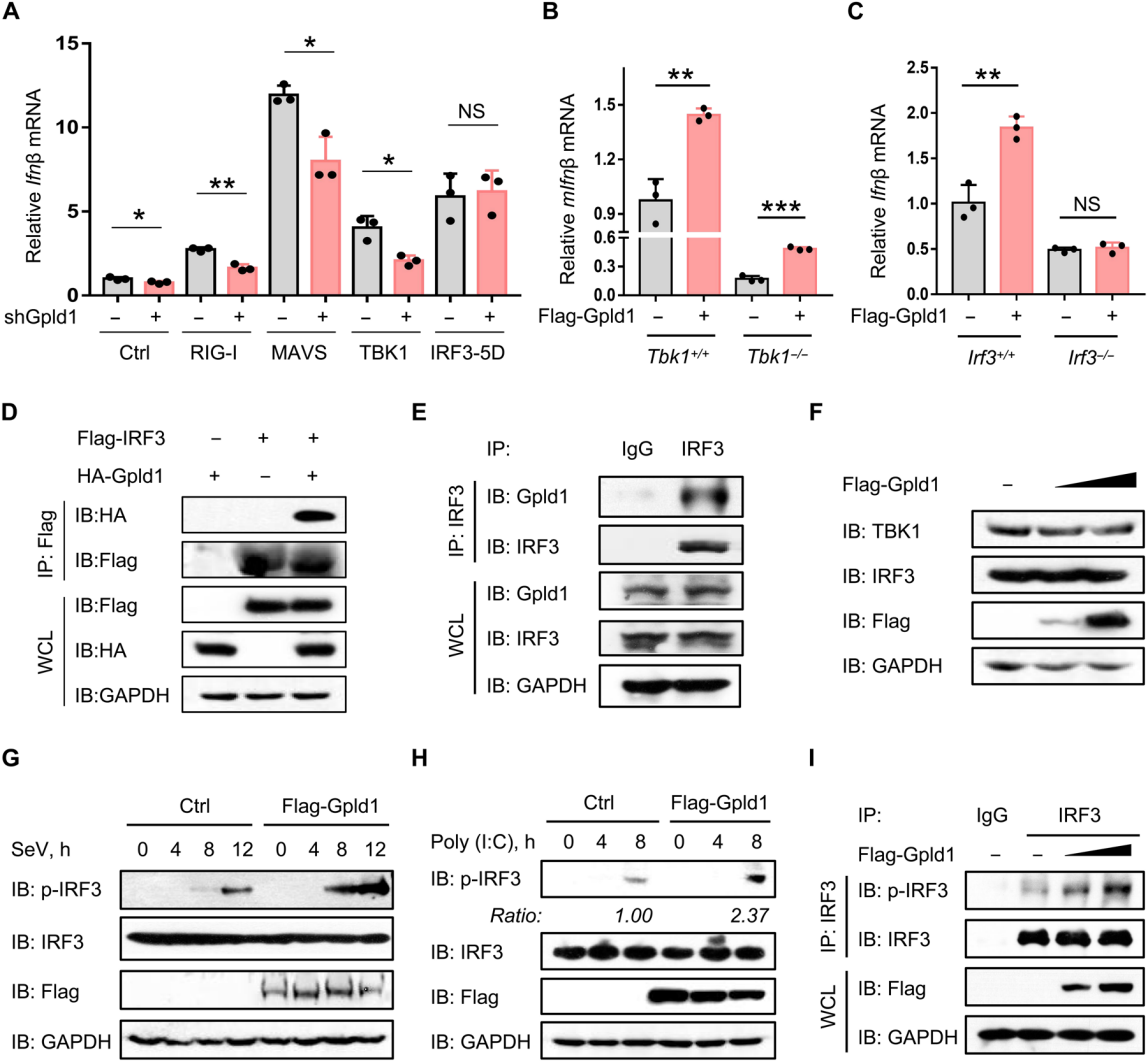
Gpld1 targets IRF3 and promotes IRF3 phosphorylation. (**A**) RT-qPCR analysis of *Ifn*β mRNA in HEK293T cells cotransfected with or without Flag-tagged RIG-I/MAVS/TBK1/IRF3-5D and shGpld1. (**B**) RT-qPCR analysis of *mIfn*β mRNA in *Tbk1^+/+^* and *Tbk1^−/−^* MEF cells transfected with control vectors or Flag-Gpld1. (**C**) RT-qPCR analysis of *Ifn*β mRNA in *Irf3^+/+^* and *Irf3^−/−^* HEK293T cells transfected with control vectors or Flag-Gpld1. (**D**) Immunoprecipitation (IP) analysis of the interaction between Gpld1 and IRF3 in HEK293T cells cotransfected with HA-Gpld1 and Flag-IRF3. (**E**) IP analysis of the interaction between endogenous Gpld1 and IRF3 in HepG2 cells. (**F**) Western blot analysis of TBK1 and IRF3 in HEK293T cells transfected with increasing amounts of Flag-Gpld1. (**G**) Western blot analysis of phosphorylated IRF3 at the Ser^396^ residue (p-IRF3) in HEK293T cells transfected with Flag-Gpld1 and then infected with SeV (MOI = 1.0) as indicated. (**H**) Western blot analysis of p-IRF3 in HEK293T cells transfected with Flag-Gpld1 and then transfected with polyinosinic:polycytidylic acid [poly(I:C)] (1 μg/ml) as indicated. (**I**) IP analysis of p-IRF3 in HEK293T cells transfected with increasing amounts of Flag-Gpld1. NS, not significant (*P* > 0.05). *P < 0.05, **P < 0.01, and ***P < 0.001 (two-tailed unpaired Student’s *t* test). Data are shown as the mean and SD of three biological replicates [(A) to (C)], or are representative of three independent experiments [(D) to (I)].

Given that Gpld1 regulates the RNA virus VSV-induced IFN-I production, we further analyzed whether Gpld1 promotes IFN-I production through the RIG-I–like receptor signaling pathway. We found that Gpld1 knockdown inhibited IFN-I expression activated by RIG-I, mitochondrial antiviral signaling protein (MAVS), or TANK-binding kinase 1 (TBK1) but not by a constitutively active IRF3 variant IRF3-5D (Fig. 3A). In addition, Gpld1 can still regulate IFN-I production in *Tbk1^−/−^* cells (Fig. 3B and fig. S3A). IRF3 knockout abolished Gpld1-mediated regulation of IFN-I production (Fig. 3C and fig. S3B). Furthermore, we found that hemagglutinin (HA)–tagged Gpld1 can interact with exogenously expressed IRF3 (Fig. 3D) but not TBK1 (fig. S3C), and endogenous Gpld1 also interacts with cellular IRF3 (Fig. 3E).

Next, we found that Gpld1 did not affect the protein levels of both TBK1 and IRF3 (Fig. 3F and fig. S3D). Gpld1 overexpression promoted virus-induced phosphorylation of IRF3 (Fig. 3G). Similarly, polyinosinic:polycytidylic acid [poly(I:C)]–induced IRF3 phosphorylation was also up-regulated by Gpld1 (Fig. 3H). In general, basal levels of phosphorylated IRF3 and TBK1 induced by secreted endogenous IFN-I are too low to be detected by immunoblotting. Thus, we used immunoprecipitation and found that overexpression of Gpld1 substantially up-regulated basal levels of IRF3 phosphorylation (Fig. 3I) but not TBK1 phosphorylation (fig. S3E), which is consistent with the above result showing that Gpld1 overexpression up-regulated basal levels of IFN-I. Together, our findings suggested that Gpld1 interacts with IRF3 and enhances IRF3 phosphorylation to promote IFN-I production.

### Gpld1 blocks the binding of the phosphatase PP2A to IRF3

**Fig. 4.**
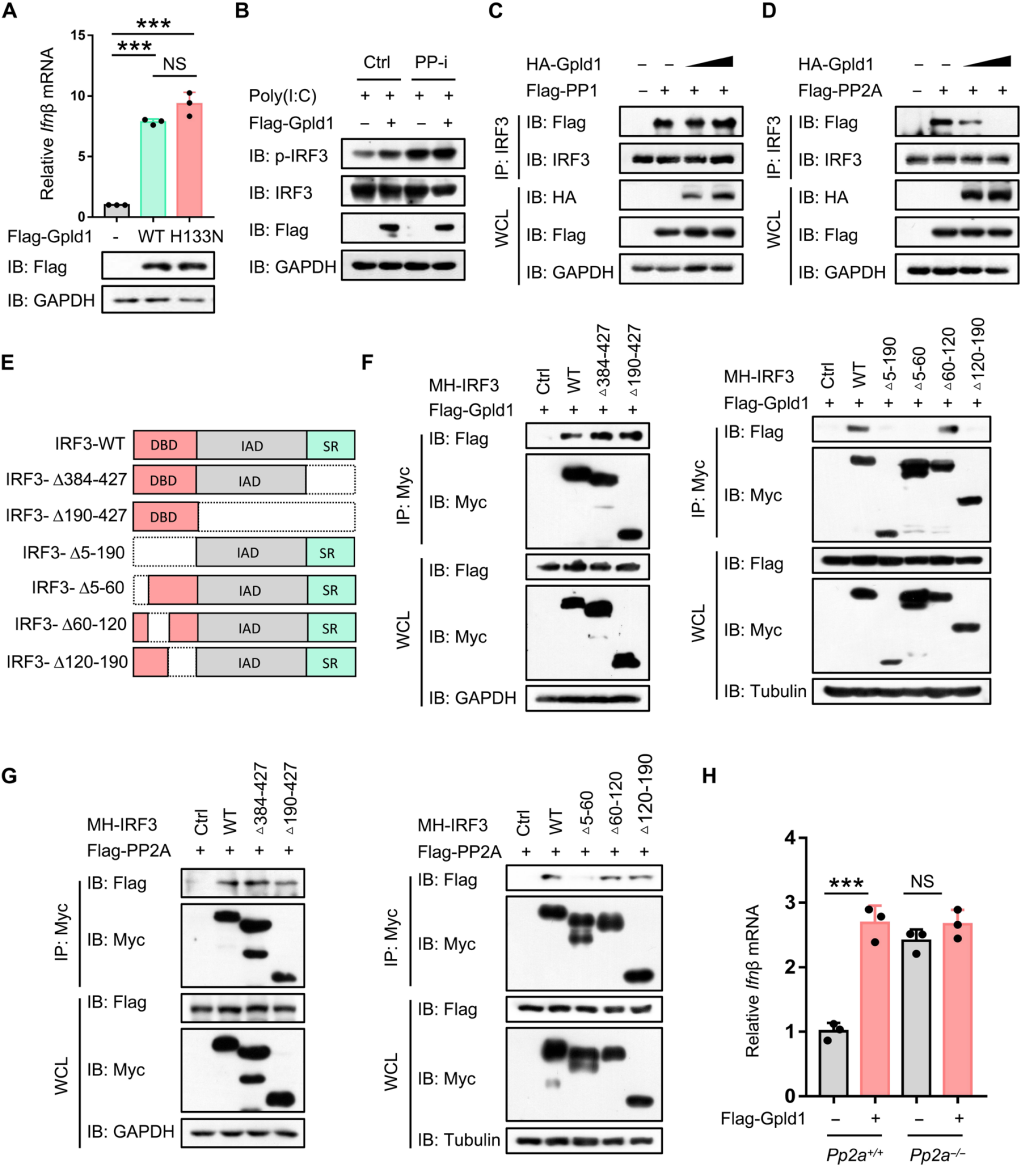
Gpld1 blocks the binding of the phosphatase PP2A to IRF3. (**A**) RT-qPCR analysis of *Ifn*β mRNA in HEK293T cells transfected with control vectors (−) or Flag-Gpld1 (WT or H133N). (**B**) Western blot analysis of p-IRF3 in 2fTGH cells transfected with Flag-Gpld1 and then treated with the phosphatase inhibitor cocktail (PP-i, Bimake), followed by transfection with poly(I:C) (1 μg/ml) for 8 hours. (**C**) IP analysis of the interaction between PP1 and IRF3 in HEK293T cells cotransfected with HA-Gpld1 and Flag-PP1. (**D**) IP analysis of the interaction between PP2A and IRF3 in HepG2 cells cotransfected with HA-Gpld1 and Flag-PP2A. (**E** and **F**) IRF3 deletion mutants were constructed (E). IP was used to analyze the IRF3 domains interacting with Gpld1 in HEK293T cells cotransfected with Flag-Gpld1 and Myc-His (MH)-tagged IRF3 (WT or mutants) (F). (**G**) IP analysis of the IRF3 domains interacting with PP2A in HEK293T cells cotransfected with Flag-PP2A and MH-IRF3 (WT or mutants). (**H**) RT-qPCR analysis of *Ifn*β mRNA in *Pp2a^+/+^* and *Pp2a^−/−^* HEK293T cells transfected with control vectors or Flag-Gpld1. NS, not significant (*P* > 0.05). ***P < 0.001 (two-tailed unpaired Student’s *t* test). Data are shown as the mean and SD of three biological replicates [(A) and (H)], or are representative of three independent experiments [(B) to (D), (F), and (G)].

We next explored the mechanism by which Gpld1 regulates IRF3 activation. It has been reported that mutation of the histidine (H) 133 residue to asparagine (N) of Gpld1 (Gpld1-H133N) resulted in a loss of its phospholipase activity (*19*). Unexpectedly, Gpld1-H133N was still able to promote IFN-I production with the same efficiency as wild-type (WT) Gpld1 (Fig. 4A), suggesting that Gpld1-mediated regulation of IRF3 activation is independent of the phospholipase activity of Gpld1. In addition, considering that AKT3 is also directly involved in IRF3 activation (*25*), we blocked AKT3 activity using the inhibitor LY294002. The results showed that Gpld1 can still up-regulate virus-induced IRF3 activation (fig. S4A).

Next, we used different types of the phosphatase inhibitor cocktail to observe whether the phosphatases are involved in Gpld1-mediated IRF3 activation. The results showed that the phosphatase inhibitors abolished Gpld1-mediated up-regulation of IRF3 activation (Fig. 4B and fig. S4B), suggesting that Gpld1 regulates IRF3 activation through the phosphatases. Thus, we further analyzed two reported phosphatases of IRF3, including protein phosphatase 1 (PP1) and PP2A (*26*, *27*). We found that Gpld1 can inhibit the interaction of IRF3 with PP2A but not with PP1 (Fig. 4, C and D, and fig. S4C). To provide more evidence to demonstrate Gpld1-mediated competitive inhibition of the IRF3-PP2A interaction, we first constructed two deletion mutants of IRF3, including IRF3-Δ(190–427) (deletion of amino acids 190 to 427) and IRF3-Δ(384–427) (deletion of amino acids 384 to 427) (Fig. 4E). We observed that these two IRF3 mutants can still interact with Gpld1 (Fig. 4F, left). Thus, we further constructed other deletion mutants of IRF3, including IRF3-Δ(5–60), IRF3-Δ(60–120), IRF3-Δ(120–190), and IRF3-Δ(5–190). We found that the deletion of either amino acids 5 to 60 or 120 to 190 of IRF3 inhibited the IRF3-Gpld1 interaction (Fig. 4F, right, and fig. S4D). Furthermore, the interaction between these IRF3 mutants and PP2A was analyzed. The deletion of amino acids 5 to 60 of IRF3 also inhibited the IRF3-PP2A interaction (Fig. 4G). These interaction analyses suggested that Gpld1 and PP2A could competitively bind to the 5 to 60 amino acid region of IRF3. We found that knockout of PP2A promoted IFN-I production and blocked Gpld1-mediated up-regulation of IFN-I production (Fig. 4H and fig. S4E). Together, these findings revealed an undescribed function of Gpld1 independently of its phospholipase activity and suggested that Gpld1 inhibits PP2A-mediated dephosphorylation of IRF3 to promote IRF3 activation and IFN-I production.

### Exercise increases the metabolite 3-HB that can promote Gpld1 expression

**Fig. 5.**
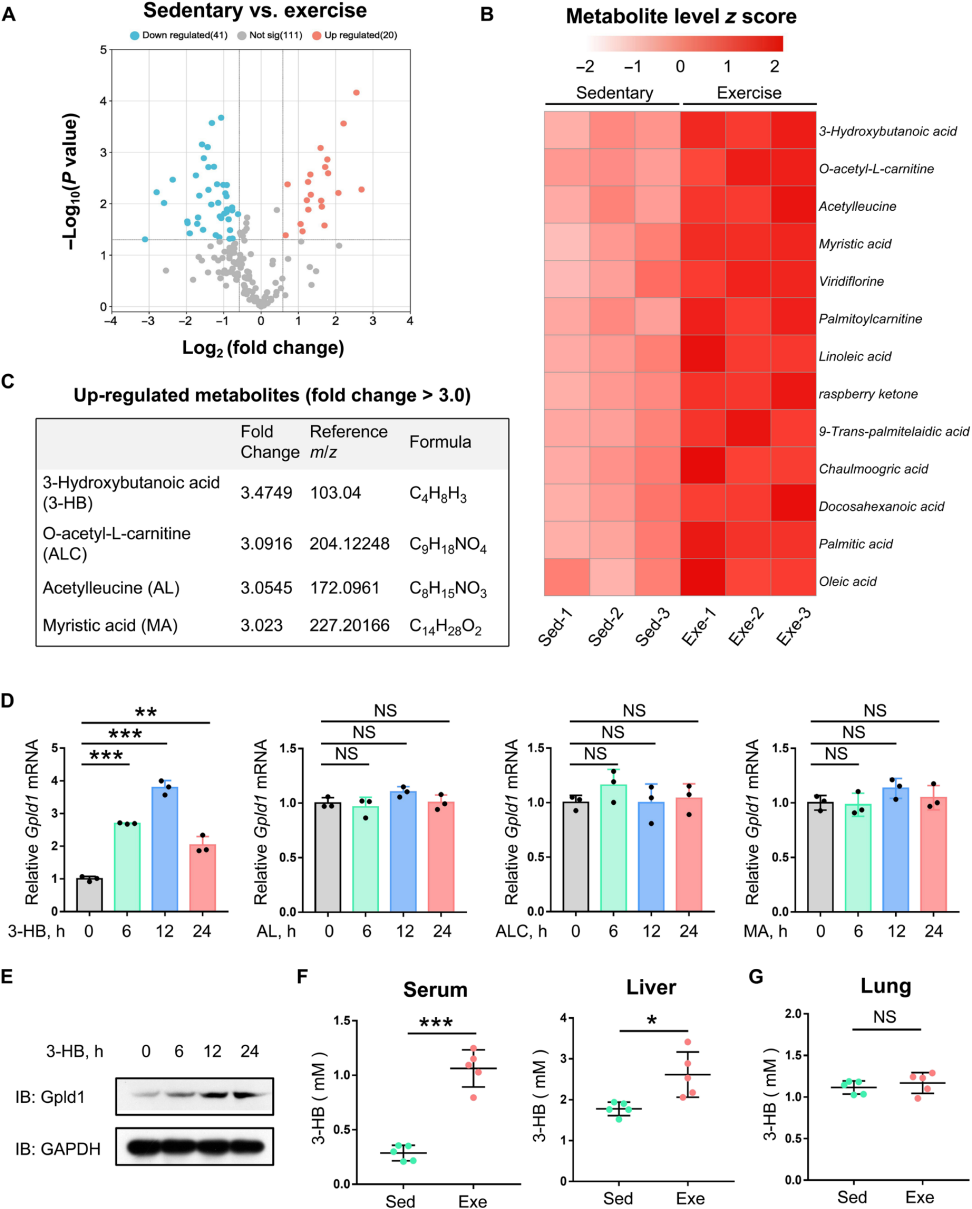
Exercise increases the metabolite 3-HB that can promote Gpld1 expression. (**A**) Untargeted metabolomics analysis of differential metabolites in the serum of sedentary mice (*n* = 3) and exercise mice (1 Wk; *n* = 3). A total of 41 metabolites were significantly down-regulated (blue), and 20 metabolites were significantly up-regulated (red) in exercised mice compared with sedentary mice. Differential metabolites were defined as the metabolites with changes ≥1.5-fold and *P* values <0.05 between two groups. (**B** and **C**) The heatmap shows top 13 metabolites that are up-regulated in the serum of exercised mice after removing those metabolites from plants and microorganisms (B). The table shows the metabolites from (B) with fold change >3.0 and *P* values <0.05 between the two groups (C). (**D**) RT-qPCR analysis of *Gpld1* mRNA in HepG2 cells treated with 3-HB (0.5 mM), AL (2.5 mM), ALC (10 μM), or MA (50 μM) as indicated. (**E**) Western blot analysis of Gpld1 protein in HepG2 cells treated with 3-HB (0.5 mM) as indicated. (**F** and **G**) Colorimetric assay analysis of 3-HB in the serum and livers (F) and the lungs (G) of sedentary mice (*n* = 5) and exercised mice (1 Wk; *n* = 5). NS, not significant (*P* > 0.05). *P < 0.05, **P < 0.01, and ***P < 0.001 (two-tailed unpaired Student’s *t* test). Data are shown as the mean and SD of three biological replicates (D) or five individual mice [(F) and (G)], or are representative of three independent experiments (E).

As mentioned above, Gpld1 promotes IFN-I production in the liver after exercise, while exercise strongly accelerates metabolism and the liver is a crucial organ of metabolism of the body. Thus, we next determined whether certain metabolites could be involved in the up-regulation of Gpld1. To this end, we performed untargeted metabolomics analyses of differential metabolites in the serum from exercised or sedentary mice (Fig. 5, A and B). We observed 41 down-regulated metabolites and 20 up-regulated metabolites in the serum of exercised mice, compared with those of sedentary mice (Fig. 5A). After excluding the metabolites only from plants and microorganisms, we got 13 significantly up-regulated metabolites (Fig. 5B). We further selected the metabolites showing a fold change >3.0 after exercise, including 3-HB, O-acetyl-l-carnitine (ALC), acetylleucine (AL), and myristic acid (MA) (Fig. 5C). We found that 3-HB but not the other three metabolites significantly up-regulated the mRNA levels of Gpld1 (Fig. 5D). Meanwhile, 3-HB promoted Gpld1 expression in a dose-dependent manner (fig. S5A). Consistently, Gpld1 protein levels were up-regulated by only 3-HB but not AL, ALC, or MA (Fig. 5E and fig. S5, B to D). These findings suggested that 3-HB is able to promote Gpld1 transcriptional expression. We found that the metabolite 3-HB was significantly up-regulated in both the serum and the livers of mice with 1 week of exercise (Fig. 5F), while exercise did not significantly up-regulate 3-HB levels in the lung (Fig. 5G). Collectively, these findings suggested that exercise up-regulates the metabolite 3-HB that can significantly promote Gpld1 expression in the liver.

### 3-HB is a positive regulator of both IFN-I production and cellular antiviral activity

**Fig. 6.**
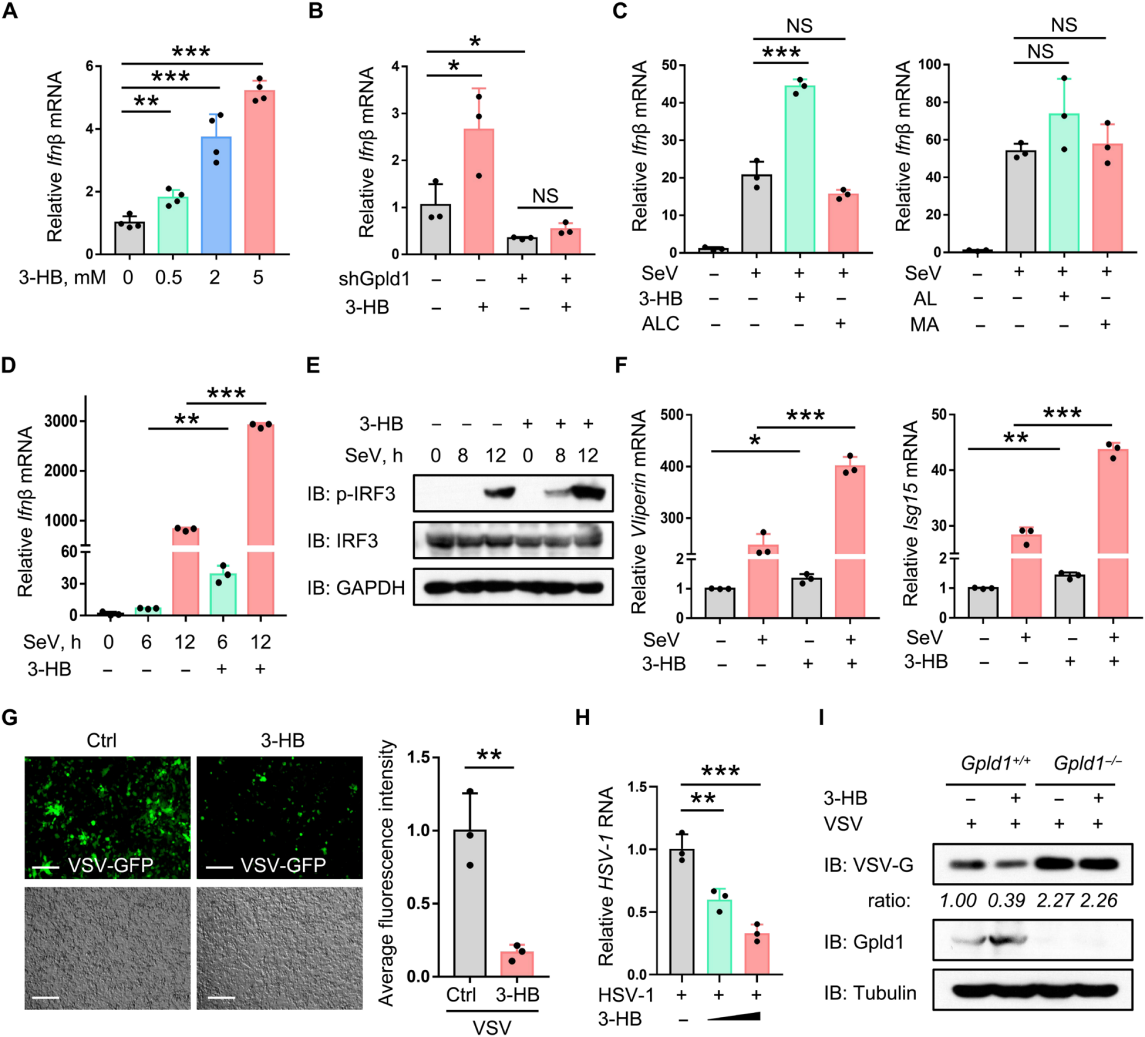
3-HB is a positive regulator of both IFN-I production and cellular antiviral activity. (**A**) RT-qPCR analysis of *Ifn*β mRNA in HepG2 cells treated with 3-HB for 24 hours as indicated. (**B**) RT-qPCR analysis of *Ifn*β mRNA in HepG2 cells transfected with shCtrl or shGpld1 and then treated with 3-HB (0.5 mM; 24 h). (**C**) RT-qPCR analysis of *Ifn*β mRNA in HepG2 cells treated with 3-HB (5 mM), ALC (10 μM), AL (2.5 mM), or MA (50 μM) for 24 hours and then infected with SeV (MOI = 1.0; 12 hours). (**D**) RT-qPCR analysis of *Ifn*β mRNA in HepG2 cells treated with 3-HB (5 mM) for 24 hours and then infected with SeV (MOI = 1.0) as indicated. (**E**) Western blot analysis of p-IRF3 in HepG2 cells treated with 3-HB (5 mM; 24 hours) and then infected with SeV as (D). (**F**) RT-qPCR analysis of *Viperin* and *Isg15* mRNA in HepG2 cells treated with 3-HB as (D) and then infected with SeV (MOI = 1.0; 12 hours). (**G**) Fluorescence microscopy of VSV-GFP viruses in HepG2 cells treated with PBS (Ctrl) or 3-HB (5 mM) for 24 hours and then infected with VSV-GFP (MOI = 1.0; 24 hours). Scale bars, 200 μm. (**H**) RT-qPCR analysis of HSV-1 RNA in HepG2 cells pretreated with 3-HB (5 mM; 24 hours) and then infected with HSV-1 (MOI = 1.0; 24 hours). (**I**) Western blot analysis of VSV-G in *Gpld1^+/+^* or *Gpld1^−/−^* HEK293T cells pretreated with 3-HB (5 mM; 24 hours) and then infected with VSV (MOI = 1.0; 24 hours). NS, not significant (*P* > 0.05). *P < 0.05, **P < 0.01, and ***P < 0.001 (two-tailed unpaired Student’s *t* test). Data are shown as mean and SD of three biological replicates [(A) to (D), (F), and (H)], or are representative of three independent experiments [(E) and (I)].

Given that 3-HB promotes Gpld1 expression and Gpld1 up-regulates IFN-I production, we speculated that 3-HB could regulate IFN-I antiviral response. We found that 3-HB treatment did up-regulate the levels of IFN-I mRNA in a dose-dependent manner (Fig. 6A), while knockdown of Gpld1 blocked 3-HB–induced up-regulation of IFN-I (Fig. 6B). Furthermore, 3-HB but not ALC, AL, or MA promoted virus-induced IFN-I production (Fig. 6, C and D). Consistently, 3-HB can promote virus-induced IRF3 phosphorylation during virus infection (Fig. 6E). In addition, 3-HB enhanced the expression of ISGs during virus infection (Fig. 6F and fig. S6A). In line with ISG up-regulation, 3-HB treatment lowered the levels of viruses in cells infected with VSV, HSV-1, or HBV (Fig. 6, G and H, and fig. S6B). Gpld1 knockout largely inhibited 3-HB–mediated inhibition of virus infection (Fig. 6I and fig. S6C). Thus, we demonstrated that 3-HB positively regulates IRF3 activation, IFN-I production, and IFN-I antiviral activity.

### 3-HB promotes Gpld1 expression in the liver and improves IFN-I antiviral immunity in vivo

**Fig. 7.**
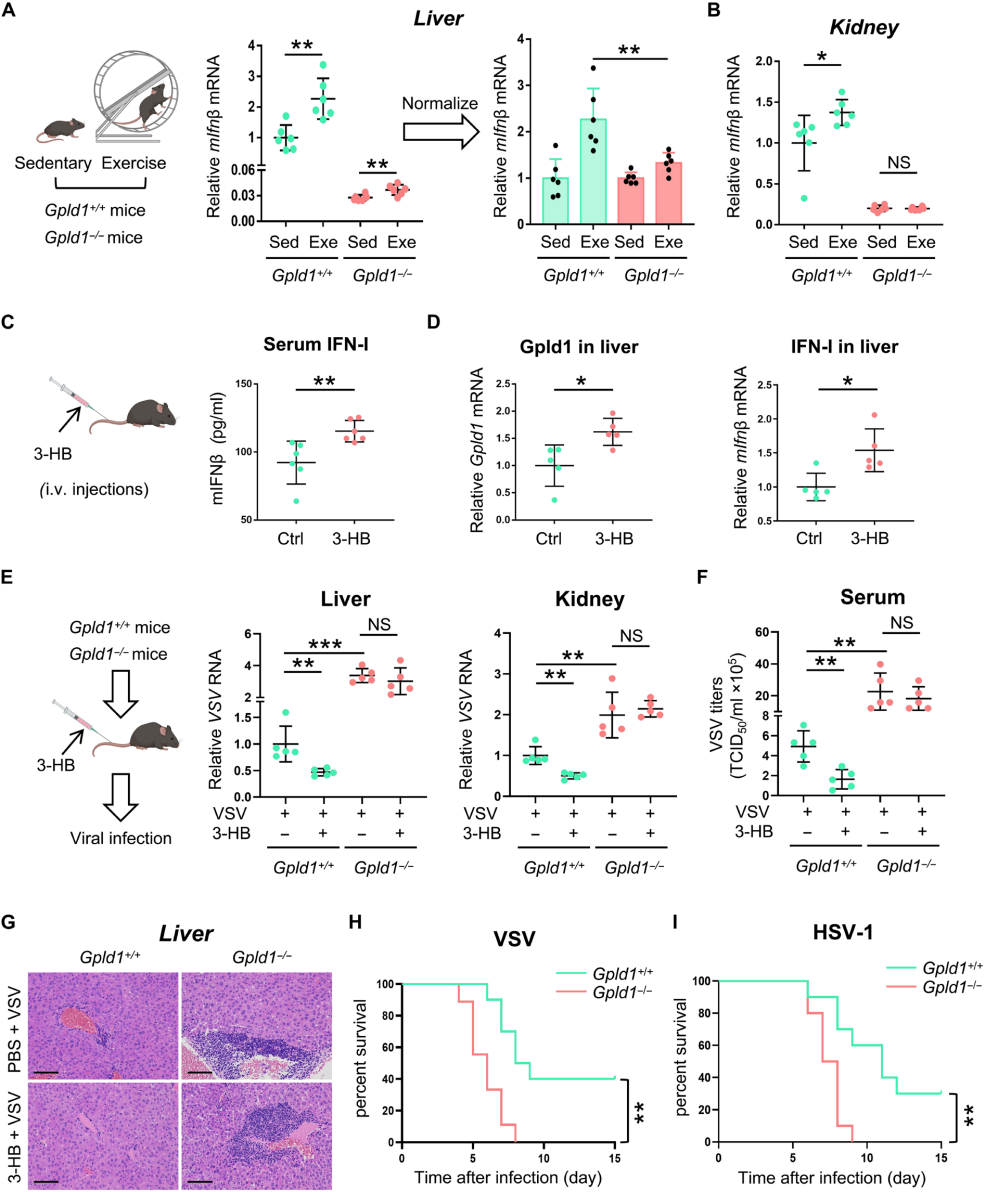
3-HB promotes Gpld1 expression in the liver and improves IFN-I antiviral immunity in vivo. (**A**) RT-qPCR analysis of *mIfn*β mRNA levels in the livers of *Gpld1^+/+^* (*n* = 6) and *Gpld1^−/−^* (*n* = 6) mice with (Exe) or without (Sed) 1 week of exercise (left). The efficiency of *mIfn*β induction in exercised mice was further assessed by normalizing to that in sedentary *Gpld1^+/+^* or sedentary *Gpld1^−/−^* mice respectively (right). This model in (A) was created with BioRender.com. (**B**) RT-qPCR analysis of *mIfn*β mRNA levels in the kidneys from (A). (**C**) ELISA analysis of mIFN-β protein levels in the serum of mice administrated with 3-HB (3 mmol/kg, i.v.) or PBS (Ctrl) for 24 hours. This model in (C) was created with BioRender.com. (**D**) RT-qPCR analysis of *Gpld1* mRNA or *mIfn*β mRNA in the livers from (C). (**E**) RT-qPCR analysis of the livers and kidneys of *Gpld1*^+/+^ and *Gpld1*^−/−^ mice administrated with 3-HB (3 mmol/kg, i.v.) or PBS for 24 hours, and then infected with VSV (1 × 10^8^ PFU/g of body weight; intranasal) for 48 hours. This model in (E) was created with BioRender.com. (**F**) TCID_50_ assay of virus titers in the serum of from (E). (**G**) HE staining of the livers from (E). Scale bars, 100 μm. (**H** and **I**) Survival curves of *Gpld1*^+/+^ and *Gpld1*^−/−^ mice infected with VSV (1 × 10^8^ PFU/g body weight, *n* = 10, i.v.) (H) or HSV-1 (2 × 10^6^ PFU/g body weight, *n* = 10, i.v.) (I). NS, not significant (*P* > 0.05). *P < 0.05, **P < 0.01, and ***P < 0.001 (two-tailed unpaired Student’s *t* test). Data are shown as mean and SD of at least five individual mice [(A) to (F)].

We further observed the in vivo effects of both Gpld1 and 3-HB on IFN-I production and host antiviral response. We found that IFN-I levels in both the livers and the kidneys of *Gpld1^−/−^* mice were significantly lower than those of *Gpld1^+/+^* mice (Fig. 7, A and B), confirming that Gpld1 is a positive regulator of IFN-I production in the body. Exercise-induced IFN-I production was largely attenuated in the livers of *Gpld1^−/−^* mice (Fig. 7A) and was even abolished in the kidneys of *Gpld1^−/−^* mice (Fig. 7B). Gpld1 deficiency did not block exercise-induced up-regulation of 3-HB levels (fig. S7A). These findings suggested that exercise-induced IFN-I production is largely mediated by Gpld1, although other exercise-induced factors could be involved in IFN-I production more or less.

Next, we injected 3-HB into the mice intravenously (i.v.) via tail vein. We found that 3-HB administration up-regulated serum IFN-I levels (Fig. 7C). Furthermore, we analyzed Gpld1 expression and IFN-I production in the livers of mice with 3-HB administration. The results showed that 3-HB administration promoted Gpld1 expression in the livers of mice (Fig. 7D, left). Consistently, IFN-I levels in the livers were also up-regulated by 3-HB (Fig. 7D, right). We noticed that both Gpld1 and IFN-I levels were also slightly up-regulated in the kidneys of mice with 3-HB administration (fig. S7B). In contrast, Gpld1 and IFN-I levels in the spleens and lungs were not significantly up-regulated after 3-HB treatment (fig. S7, C and D). Next, we determined the in vivo effect of 3-HB on antiviral immune response. The results showed that 3-HB administration substantially lowered virus RNA levels in the livers, kidneys, and lungs of *Gpld1^+/+^* mice (Fig. 7E and fig. S7E). Gpld1 knockout abolished 3-HB–mediated inhibition of virus infection (Fig. 7E and fig. S7E). Consistently, 3-HB administration also reduced the virus titers in the serum of *Gpld1^+/+^* mice, while this antiviral effect was abolished by Gpld1 knockout (Fig. 7F). Furthermore, hematoxylin and eosin (HE) staining confirmed 3-HB–mediated inhibition of virus infection in vivo (Fig. 7G and fig. S7F). Moreover, Gpld1 deficiency significantly lowered the survival rate of mice in response to infection with either VSV (Fig. 7H) or HSV-1 (Fig. 7I). Together, these findings suggested that 3-HB promotes Gpld1 expression in vivo and enhances IFN-I antiviral immunity of the body.

## DISCUSSION

Daily behavioral patterns are closely related to people’s health status. Although most of people think that the exercise behavior may enhance immunity of the body, the specific contents of exercise-regulated immune activity and the underlying mechanisms are still unclear. In this study, we focused on exploring whether and how exercise regulates antiviral innate immunity of the body. Actually, the difference in the intensity, duration, and regularity of exercise may lead to completely different consequences for immune regulation. Moderate exercise has been reported to help to defend against virus infection, but high-intensity exercise could be counterproductive (*4*, *28*–*30*). Therefore, we chose to allow the mice to exercise autonomously and regularly (three times a day, 1 hour each time, and for a total of 1 day or 1 week). We observed that IFN-I was significantly up-regulated in the serum and the liver, and slightly up-regulated in the kidneys of mice with 1 week of moderate exercise. However, 1 day of exercise did not promote IFN-I production, suggesting that an occasional exercise could not have a significant effect on the enhancement of IFN-I response. This study revealed that exercise can promote IFN-I immune response.

The liver is one of the major metabolic organs, where most of the metabolic activities occur. Exercise leads to high metabolism, which may change the expression of many proteins in the liver. Here, our study showed that exercise promoted IFN-I production in the liver, and the liver is the major organ that results in an increase in serum IFN-I levels after exercise. Therefore, we next determined which proteins are strongly up-regulated in the liver and majorly contribute to IFN-I production in the liver after exercise. Recently, a comprehensive study has identified the exercise-induced proteins that are mostly of hepatic origin (*19*). Consistent with this report, we also observed the up-regulation of Gpld1 expression after exercise. We further demonstrated that Gpld1 critically promotes IFN-I production and exerts antiviral immune in the liver. Unexpectedly, Gpld1 enhances the expression of IFN-I in an enzyme activity–independent manner. Together, these findings uncovered a role of the liver in antiviral innate immunity and identified an undescribed activity of Gpld1 independently of the phospholipase activity.

Given that the liver is one of the main organs of metabolism, we speculated that certain metabolites could contribute to Gpld1 expression after exercise. Thus, we performed an untargeted metabolomic analysis of the serum from exercised and sedentary mice. We then identified 3-HB as an important metabolite that was up-regulated in the liver after exercise and significantly promoted Gpld1 expression in a dose-dependent manner. It is noteworthy that 3-HB is mainly derived from fatty acid oxidation in the liver. Therefore, we hypothesized that exercise could exacerbate hepatic fatty acid oxidation, leading to up-regulation of 3-HB. 3-HB in the liver in turn promotes Gpld1 expression, while those 3-HB released into the serum from the liver could enter the other tissues such as the kidney to induce Gpld1 expression to some extent. Thus, this study identified 3-HB as an unreported metabolite that regulates antiviral innate immunity.

However, the pathways through which 3-HB promotes Gpld1 expression remain to be investigated. To date, although several receptors of 3-HB, as well as transporter proteins for 3-HB, have been reported, 3-HB itself actually has good cell membrane permeability (*31*–*34*). Therefore, 3-HB could enter the cells in various ways and then act as a regulator to activate the transcription of Gpld1. In addition, the concentration of 3-HB in human blood fluctuates widely and is regulated by a variety of factors. The basal level of 3-HB in human serum is relatively low. However, it can reach 1 to 2 mM after 90 min of strenuous exercise. Moreover, the ketogenic diet can also make 3-HB levels rise to more than 2 mM, and 3-HB can even reach 6 to 8 mM after prolonged starvation (*35*–*37*). Comparably, the 3-HB concentration in our study ranged from 0.5 to 5 mM, and we found that 3-HB up-regulates Gpld1 in a time-dependent and dose-dependent manner. According to these findings, we think that it could be feasible to use 3-HB for the development of antiviral therapeutic strategies in the future.

**Fig. 8.**
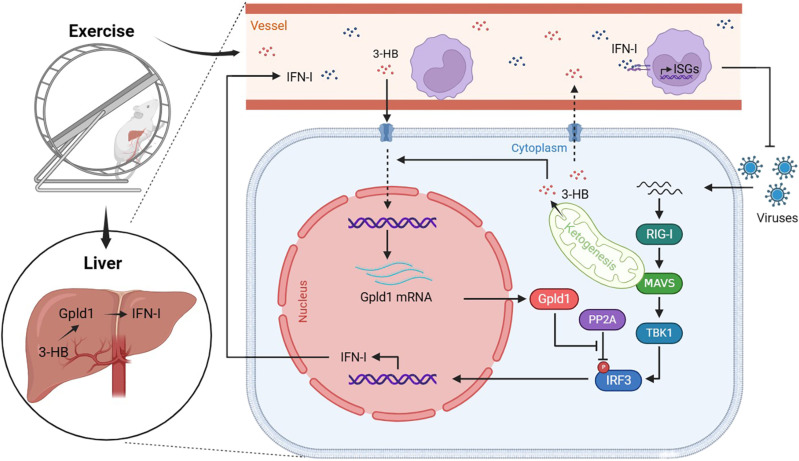
A proposed model of exercise to enhance antiviral immunity. Exercise leads to an increase in 3-HB that enhances Gpld1 expression in the liver. Gpld1 inhibits the interaction between the phosphatase PP2A and IRF3, thus promoting IFN production and secretion from the liver, eventually improving serum IFN levels and antiviral immunity of the body. This model was created with BioRender.com.

Gpld1 may perform different functions in different types of cells. Gpld1 has been showed to be predominantly expressed in the liver, with varying degrees of expression in other organs (*38*). Here, our study suggested that 3-HB administration up-regulates Gpld1 levels mainly in the liver but not in the spleens and lungs. It has been reported that 3-HB is mainly synthesized in the liver, and the kidney is also involved in synthesizing a small amount of 3-HB (*22**,* *39*). In addition, on the basis of the National Center for Biotechnology Information (NCBI) database, we noticed that 3-hydroxy-3-methylglutaryl-CoA synthase 2, a rate-limiting enzyme for 3-HB synthesis (*21*), is also highly expressed in the liver and kidney, with the highest expression in the liver. Therefore, we hypothesized that exercise leads to increased synthesis of 3-HB mainly in the liver, which promotes Gpld1 expression and subsequent IFN-I production and secretion in the liver, thus eventually improving IFN-I–mediated antiviral immunity of the whole body (Fig. 8).

In summary, this study revealed a 3-HB-Gpld1-IFN signaling axis in the liver, which links exercise, metabolism, and antiviral innate immunity of the body. These findings provide evidence for understanding of exercise-regulated immunity and could help to develop strategies for the prevention and treatment of virus-related diseases.

## MATERIALS AND METHODS

### Mice

*Gpld1*^−/−^ mice (C57BL/6) were generated by the Cyagen Bioscience Inc. (Guangzhou, China). The Gpld1 gene (NCBI reference sequence, NM_008156.2; and Ensembl, ENSMUSG00000021340) is located on mouse chromosome 13. Exons 3 to 6 are selected as the knockout target site. The size of effective knockout region is ~9171 bp. Six- to 8-week-old *Gpld1*^−/−^ mice were used for the experiments in this study. WT C57BL/6 mice were purchased from the Laboratory Animal Center of Soochow University. Mice were maintained and bred in special pathogen–free conditions in the Experimental Animal Center of Soochow University. The animal care and use protocol adhered to the National Regulations for the Administration of Affairs Concerning Experimental Animals. All protocols and procedures for mouse studies were performed in accordance with the Laboratory Animal Management Regulations with approval of the Scientific Investigation Board of Soochow University.

### Establishment of the exercised mouse model

The mice (C57BL/6) were randomly divided into a sedentary group and an exercise group. The mice in the exercised group exercised three times a day (in the morning, afternoon and evening) and 1 hour each time in the running wheel for a total of 1 day or 1 week. The mice in the sedentary group were provided with enough water and food to keep them in cages.

### Primary cells from mice

Mouse liver was prepared from the 6- to 8-week mice (WT or *Gpld1^−/−^*), and then the liver was cut into small pieces and digested with collagenase IV (Solarbio). After that, mouse primary hepatocytes were collected by centrifugation for subsequent experiments. Bone marrow was extracted from the limbs of 6- to 8-week male mice by perfusion with phosphate-buffered saline (PBS). After centrifugation, an erythrocyte lysis solution was added. Mouse bone marrow cells were obtained after centrifugation.

### Expression constructs and reagents

Human Flag-Gpld1, Flag-Bche, Flag-PP1, and Flag-PP2A were purchased from the YouBio Company. Human Flag-Pon1, Flag-Mbl2, and Flag-Itih2 were purchased from Vigene Biosciences. HA-Gpld1 was made using polymerase chain reaction (PCR) amplification from Flag-Gpld1 and cloned into the HA-pcDNA3.1 vector. GFP-N1-Gpld1 was made using PCR amplification from Flag-Gpld1 and cloned into the GFP-N1 vector. Human Flag-MAVS was a gift from C. Gao (Shandong University, China). Human Flag-RIG-I, Flag-TBK1, Flag-IRF3, HA-IRF3, and Flag-IRF3-5D were gifts from F. Zhou (Soochow University). Myc-His-IRF3 was made using PCR amplification from the HA-IRF3 plasmids and cloned into the Myc-His vector. Myc-His-IRF3 mutants (Δ5–190, Δ190–427, Δ384–427, Δ5–60, Δ60–120, and Δ120–190) were amplified using PCR from the Myc-His-IRF3 plasmid and cloned into the Myc-His vector. Flag-Gpld1-H133N was generated by a QuickChange Lightning site-Directed Mutagenesis Kit (Stratagene, 210518). shGpld1-1# (5′-GTGCCAGTCAAAGATCTAC-3′) and shGpld1-2# (5′-CTCCTATTCAGAGG CTCAT-3′) were constructed into the shX vector (gift from J. Dai, Soochow University). All plasmids were confirmed by DNA sequencing. Phosphatidylinositol 3-kinase inhibitor (LY294002) (Cayman, 70920), poly(I:C) (Sigma-Aldrich, P0913), phosphatase inhibitor cocktail (PPi) (Bimake, B15001), phosphatase inhibitor cocktail (PPi-2) (Fdbio, FD1002), and 3-HB (MedChemExpress, HY-W051723).

### Cell culture and maintenance, transfection

Human embryonic kidney 293T (HEK293T), HepG2, and Vero cells were obtained from American Type Culture Collection. 2fTGH were provided by S. Y. Fuchs (University of Pennsylvania). *Tbk1^−/−^* MEF cells were provided by J. Dai (Soochow University, China). All cells above were cultured in Dulbecco’s modified Eagle’s medium (DMEM; HyClone) supplemented with 10% fetal bovine serum (GIBCO, Life Technologies), penicillin (100 U/ml), and streptomycin (100 μg/ml). All cells were cultured at 37°C under 5% CO_2_. All transient transfections were carried out using GenePORTER2 (Genlantis, T202015) or LongTrans (Ucallm) according to the manufacturer’s instruction.

### CRISPR-Cas9–mediated genome editing

The lenti-CRISPRv2 vector was a gift from F. Zhou (Soochow University, China). For gene knockout, small guide RNAs (sgRNAs) were first cloned into the lenti-CRISPRv2 vector and were then transfected into HEK293T cells. Forty-eight hours after transfection, the cells were cultured under puromycin (1.5 μg/ml) selection for 2 weeks, and then the single clones were picked, grown, and cells were identified by immunoblotting analysis. The sgRNA sequences were as follows: human *Gpld1*, 5′-TGGAATTACTTCTCACATGG-3′; human *PP2A* (sgRNA1), 5′-ACATCGAACCTCTTGCACGT-3′; human *PP2A* (sgRNA2), 5′-GGTCAAGAGCCTCTGCGAGA-3′; human *Irf3* (sgRNA1), 5′-GCCGTAGGCCGTGCTTCCAA-3′; and human *Irf3* (sgRNA2), 5′-GCAACCCTTCTTTGCGGTTG-3′.

### Viral infection in vitro

Sendai virus (SeV) was a gift from C. Wang (China Pharmaceutical University). HSV-1 was a gift from C. Zheng (University of Calgary, Canada). Influenza A virus (H1N1, PR/8/34) was a gift from J. Dai (Soochow University, China). VSV-GFP and pUC19-HBV1.3 were gifts from C. Dong (Soochow University, China). For viral infection in vitro, the culture medium was first removed. After washing twice with PBS, the cells were infected with different viruses diluted with serum-free medium for 2 hours. Then, the supernatant was removed, and the cells were washed twice with PBS. After washing, the cells were cultured in fresh medium until harvest.

### Viral infection in vivo

The mice were injected intraperitoneally or intranasally with VSV [1 × 10^8^ plaque-forming units (PFU)/g of body weight]. After 24 or 48 hours, mouse serum, livers and kidneys were harvested and analyzed by tissue culture-infective dose (TCID_50_) assay, real-time quantitative PCR (RT-qPCR), or HE staining assay.

### TCID_50_ assay

Mice were given tail i.v. injections of 3-HB (3 mmol/kg) for 24 hours and then infected with VSV (1 × 10^8^ PFU/g of body weight) for 24 hours. The serum of mice containing VSV viruses was serially diluted with DMEM and then placed on a monolayer of Vero cells in 96-well plates. VSV viral titers were determined by a standard 50% TCID_50_ assay. The TCID_50_ was calculated by the Spearman-Karber algorithm.

### RNA and protein extraction

Total RNAs were isolated from the cells or mouse tissues using a TRIzol reagent (TAKARA). The cDNA was synthesized from 1 μg of total RNA using the RevertAid First Strand cDNA Synthesis kit (Thermo Fisher Scientific, no. K1622) and subjected to RT-qPCR with different primers in the presence of SYBR Green Supermix (Bimake) using a StepOne Plus real-time PCR system (Applied Bioscience). For protein extraction, cells or mouse tissues were harvested using Nonidet P-40 (NP-40) lysis buffer containing 150 mM NaCl, 20 mM tris-HCl (pH 7.4), 0.5 mM EDTA, 1% NP-40, phenylmethylsulfonyl fluoride (PMSF; 50 μg/ml) and protease inhibitor mixtures (Sigma-Aldrich). After centrifugation at 12,000*g* for 15 min, proteins were collected and quantified by Western blotting.

### RT-qPCR

RT-qPCR was conducted with SYBR Green (Bimake), and the relative gene expression levels were calculated using the change-in-cycling-threshold (2^−ΔΔCt^) method. Quantification of all target genes was normalized to the control gene β-actin or Gapdh, and all data are shown as fold change normalized to that in either unstimulated or uninfected cells accordingly. The results were analyzed from three independent experiments and are shown as the average mean ± SD. The primer sequences are as follows: SeV (5′-GATGACGATGCCGCAGCAGTAG-3′ and 5′-CCTCCGATGTCAGTT GGTTCACTC-3′); VSV (5′-ACGGCGTACTTCCAGATGG-3′ and 5′-CTCGGTTCAAGATCCAGG T-3′); H1N1 (5′-TTCTAACCGAGGTCGAAACG-3′ and 5′-ACAAAGCGTCTACGCTG CAG-3′); HBV (5′-TGGATTCGCACTCCTCCAGCTT-3′ and 5′-GGGACCTGCCTCGTC GTCTA-3′); HSV-*Ul42* (5′-CCAACGCCAAGACGGTGTA-3′ and 5′-GGGGGTCGTGAG GAAGAAC-3′); HSV-*Icp27* (5′-ATCGCACCTTCTCTGTGGTC-3′ and 5′-GCAAATCTTCTGGG GTTTCA-3′); human-*Ifn*β (5′-CATTACCTGAAGGCCAAGGA-3′ and 5′-CAGCATCTGCT GGTTGAAGA-3′); *Ifit1* (5′-CACAAGCCATTTTCTTTGCT-3′ and 5′-ACTTGGCTGCATATCGAA AG-3′); *Isg15* (5′-GGGACCTGACGGTGAAGATG-3′ and 5′-CGCCGATCTTCTGG GTGAT-3′); *Isg54* (5′-CACCTCTGGACTGGCAATAGC-3′ and 5′-GTCAGGATTCAGCCGA ATGG-3′); *Viperin* (5′-CCAGTGCAACTACAAATGCGGC-3′ and 5′-CGGTCTTGAAGAA ATGGCTCTCC-3′); mouse-*Ifn*β (5′-GCCTTTGCCATCCAAGAGATGC-3′ and 5′-ACACTGTCTG CTGGTGGAGTTC-3′); β*-actin* (5′-ACCAACTGGGACGACATGGAGAAA-3′ and 5′-ATAGCACAGCCT GGATAGCAACG-3′); mouse-*Gapdh* (5′-CATCACTGCCACCCAGAAGACTG-3′ and 5′-ATGCCAG TGAGCTTCCCGTTCAG-3′); human-*Gpld1* (5′-CTTCACGGTGTCACTGTGGACA-3′ and 5′-CATACACCCT CCCAAGGCTCTT-3′); and mouse-*Gpld1* (5′-GGAAGCAGAGAGGAATTGTGGC-3′ and 5′-TCCAAACCACGAGAAGTCCTCC-3′).

### Western blot

Equivalent amounts of proteins were subjected to SDS–polyacrylamide gel electrophoresis and then transferred to polyvinylidene difluoride membranes (Millipore). Membranes were blocked with 5% nonfat milk or 5% bovine serum albumin for 1 hour at room temperature and then incubated with primary antibodies (Abs) overnight at 4°C. After washing three times with TBST (1× tris-buffered saline and Tween 20), the membranes were subjected to secondary Abs [horseradish peroxidase–conjugated goat anti-rabbit or goat anti-mouse (Bioworld)] in 1% nonfat milk for 1 hour at room temperature. After washing three times with TBST, the membranes were visualized with ECL Prime (Thermo Fisher Scientific).

The Abs with the indicated dilutions were as follows: anti-Flag (Sigma-Aldrich, F7425; 1:3000), anti-HA (Abcam, ab9110; 1:3000), anti-Myc (Abmart, M20002; 1:3000), anti-GFP (Santa Cruz Biotechnology, sc-9996; 1:3000), anti-Gpld1 (Affinity, DF9750; 1:1000), anti-IRF3 (Proteintech, 11312-1-AP; 1:1000), anti-TBK1 (Cell Signaling Technology, 3504; 1:1000), anti–p-IRF3 (Ser^396^) (Cell Signaling Technology, 4947; 1:1000), anti–p-TBK1 (Ser^172^) (Cell Signaling Technology, 5483; 1:1000), anti-HA (H1N1) (Sino Biological Inc., 11684-T56; 1:1000), anti–VSV-G (Abcam, ab1874; 1:2000), anti-PP2A (PPP2CA) (Proteintech, 13482-1-AP; 1:1000), anti–β-actin (Proteintech, 66009-1-Ig; 1:1000), anti-tubulin (Proteintech, 66031-1-Ig; 1:3000), and anti-GAPDH (GoodHere Technology, AB-M-M 001; 1:3000).

### Immunoprecipitation

Cells were harvested in lysis buffer containing 150 mM NaCl, 20 mM tris-HCl (pH 7.4), 1% NP-40, 0.5 mM EDTA, PMSF (50 μg/ml), and protease inhibitor mixtures (Sigma-Aldrich). The cell lysates were incubated with specific Abs overnight on a rotor at 4°C. Protein G agarose beads (16-266; Millipore) were washed twice and then were added into the supernatant. The mixture was incubated for 3 to 4 hours on a rotor at 4°C. For immunoprecipitation of Flag/Myc/HA-tagged proteins, M2 affinity gel (A2220; Sigma-Aldrich), and Myc or HA magnetic beads (Selleck) were added to cell lysates. The lysates were rotated for 3 to 4 hours on a rotor at 4°C. After washing four times with washing buffer, the immunoprecipitates were analyzed by Western blotting.

### Enzyme-linked immunosorbent assay and colorimetric assay

The concentrations of mouse IFN-β (E-EL-M0033c; Elabscience) were measured by enzyme-linked immunosorbent assay (ELISA) kits according to the manufacturer’s instructions. The concentrations of β-hydroxybutyrate (E-BC-K785-M; Elabscience) were measured by colorimetric assay kits according to the manufacturer’s instructions.

### Liquid chromatography–mass spectrometry untargeted metabolomics analysis

The exercised mouse model was established as mentioned above. Then, the serum of mice in the exercised group and the sedentary group were harvested and sent to the BiotechPack Company (Beijing, China) for the Liquid chromatography–mass spectrometry untargeted metabolomics analysis.

### Liver or lung histology

The livers or lungs from control or 1-day-infected mice were fixed in 4% formaldehyde solution and then embedded in paraffin. Paraffin sections were stained with HE solution and then observed by light microscopy for histological changes. Magnification was ×200.

### Statistical analysis

Comparisons between different groups were analyzed using two-tailed unpaired Student’s *t* test. Values of *P* < 0.05 were considered statistically significant. *P < 0.05, **P < 0.01, and ***P < 0.001; NS, not significant.
