# Supplementary material for: Exercise activates interferon response of the liver via Gpld1 to enhance antiviral innate immunity
Source: Sci Adv. 2024 May 29;10(22):eadk5011. doi: 10.1126/sciadv.adk5011 (PMC11804790; doi:10.1126/sciadv.adk5011)
Supplement: Supplementary file 1 — Figs. S1 to S11 [file sciadv.adk5011_sm.pdf]

Science Advances
AAAS

Supplementary Materials for

Exercise activates interferon response of the liver via Gpld1 to enhance antiviral innate immunity

Tengfei Ren *et al.*

Corresponding author: Hui Zheng, huizheng@uestc.edu.cn

Sci. Adv. **10**, eadk5011 (2024)
DOI: 10.1126/sciadv.adk5011

This PDF file includes:

Figs. S1 to S7

Fig. S1

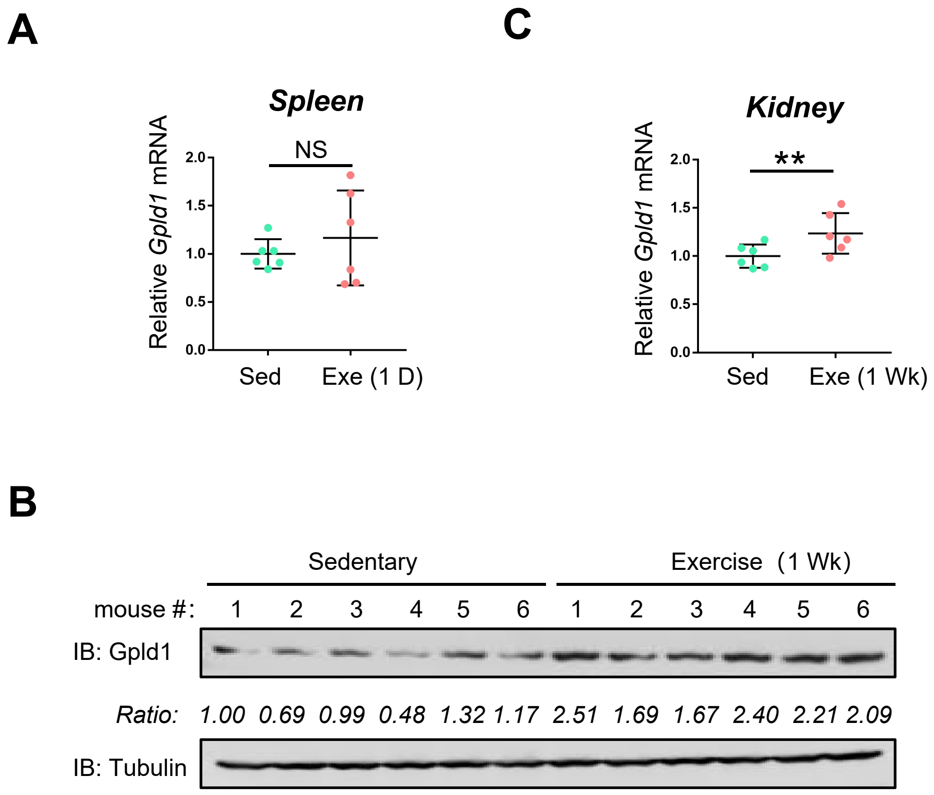

A
Spleen
NS
Relative Gpld1 mRNA
2.0
1.5
1.0
0.5
0.0
Sed
Exe (1 D)
C
Kidney
**
Relative Gpld1 mRNA
2.0
1.5
1.0
0.5
0.0
Sed
Exe (1 Wk)
B
Sedentary
Exercise (1 Wk)
mouse #: 1 2 3 4 5 6 1 2 3 4 5 6
IB: Gpld1
Ratio: 1.00 0.69 0.99 0.48 1.32 1.17 2.51 1.69 1.67 2.40 2.21 2.09
IB: Tubulin

Fig. S1. Exercise regulates the expression of Gpld1 in mouse tissues.

(A) RT-qPCR analysis of *Gpld1* mRNA levels in the spleen of sedentary (Sed) mice (n=6) and mice with one day (1 D) of exercise (Exe) (n=6) .

(B) Western blot analysis of Gpld1 protein levels in the liver of sedentary mice (n=6) and mice with one week (1 Wk) of exercise (n=6).

(C) RT-qPCR analysis of *Gpld1* mRNA levels in the kidney of sedentary mice (n=6) and exercised mice (1 Wk; n=6) .

NS, not significant ($p>0.05$). $^{**}p<0.01$ (two-tailed unpaired Student's t-test). Data are shown as the mean and SD of six individual mice (A, C), or are representative of three independent experiments (B).

Fig. S2

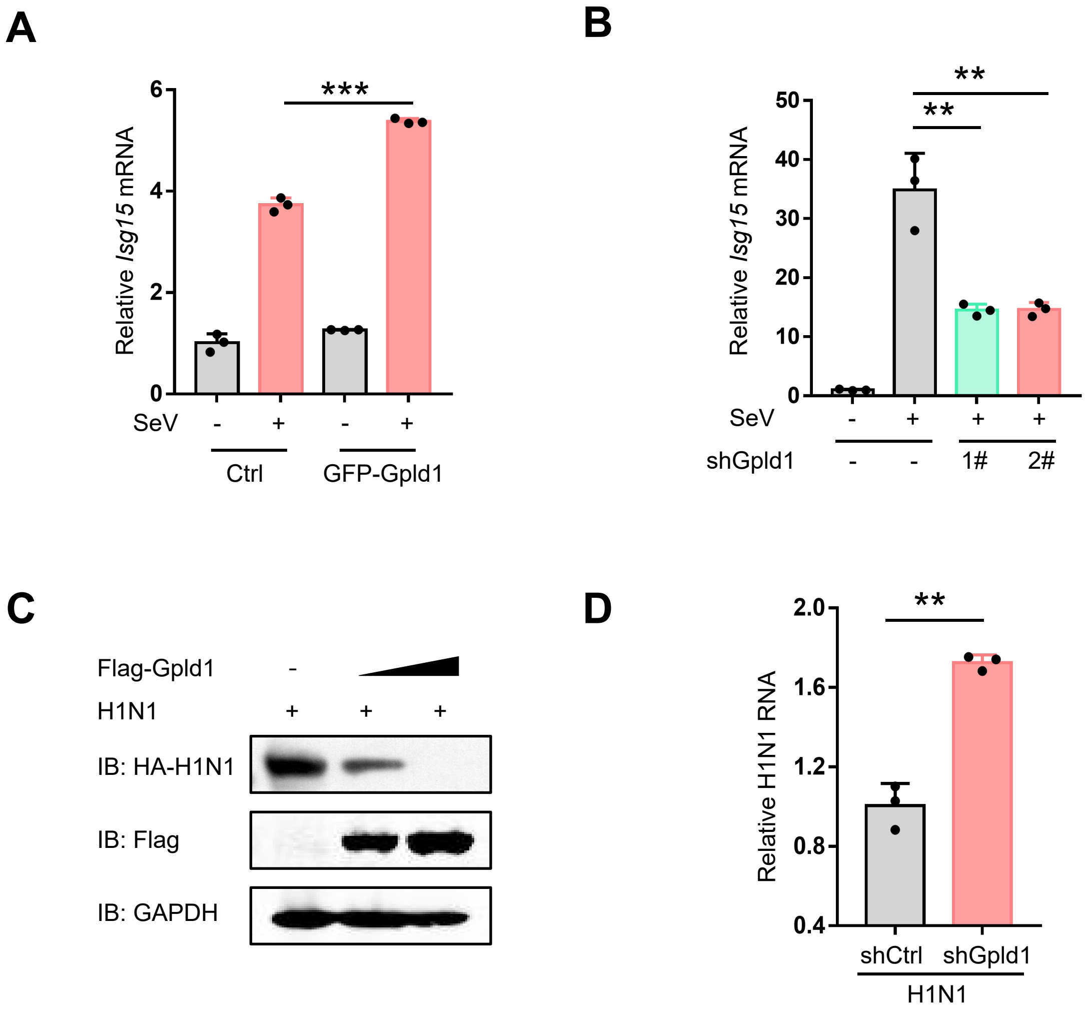
A
Relative Isg15 mRNA
6
4
2
0
***
SeV
-
+
-
+
Ctrl
GFP-Gpld1
B
Relative Isg15 mRNA
50
40
30
20
10
0
**
**
SeV
-
+
+
+
shGpld1
-
-
1#
2#
C
Flag-Gpld1
-
H1N1
+
+
+
IB: HA-H1N1
IB: Flag
IB: GAPDH
D
Relative H1N1 RNA
2.0
1.6
1.2
0.8
0.4
**
shCtrl
shGpld1
H1N1

Fig. S2. Gpld1 promotes ISGs expression and antiviral immunity.

(A) RT-qPCR analysis of *Isg15* mRNA levels in HEK293T cells transfected with the control vector (Ctrl) or GFP-Gpld1 and then infected with SeV (MOI = 1.0, 12 h) as indicated.

(B) RT-qPCR analysis of *Isg15* mRNA levels in HEK293T cells transfected with control vector or shGpld1 (1#, 2#), and then infected with SeV (MOI = 1.0, 12 h) as indicated.

(C) Western blot analysis of H1N1-encoded HA protein levels in HEK293T cells transfected with the control vector or Flag-Gpld1, and then infected with H1N1 (MOI = 1.0, 24 h) as indicated.

(D) RT-qPCR analysis of H1N1 RNA levels in HEK293T cells transfected with control shRNAs (shCtrl) or shGpld1, and then infected with H1N1 (MOI = 1.0, 24 h) as indicated.

$^{**}p<0.01$ and $^{***}p<0.001$ (two-tailed unpaired Student's t-test). Data are shown as the mean and SD of three biological replicates (A, B, D), or are representative of three independent experiments (C).

Fig. S3

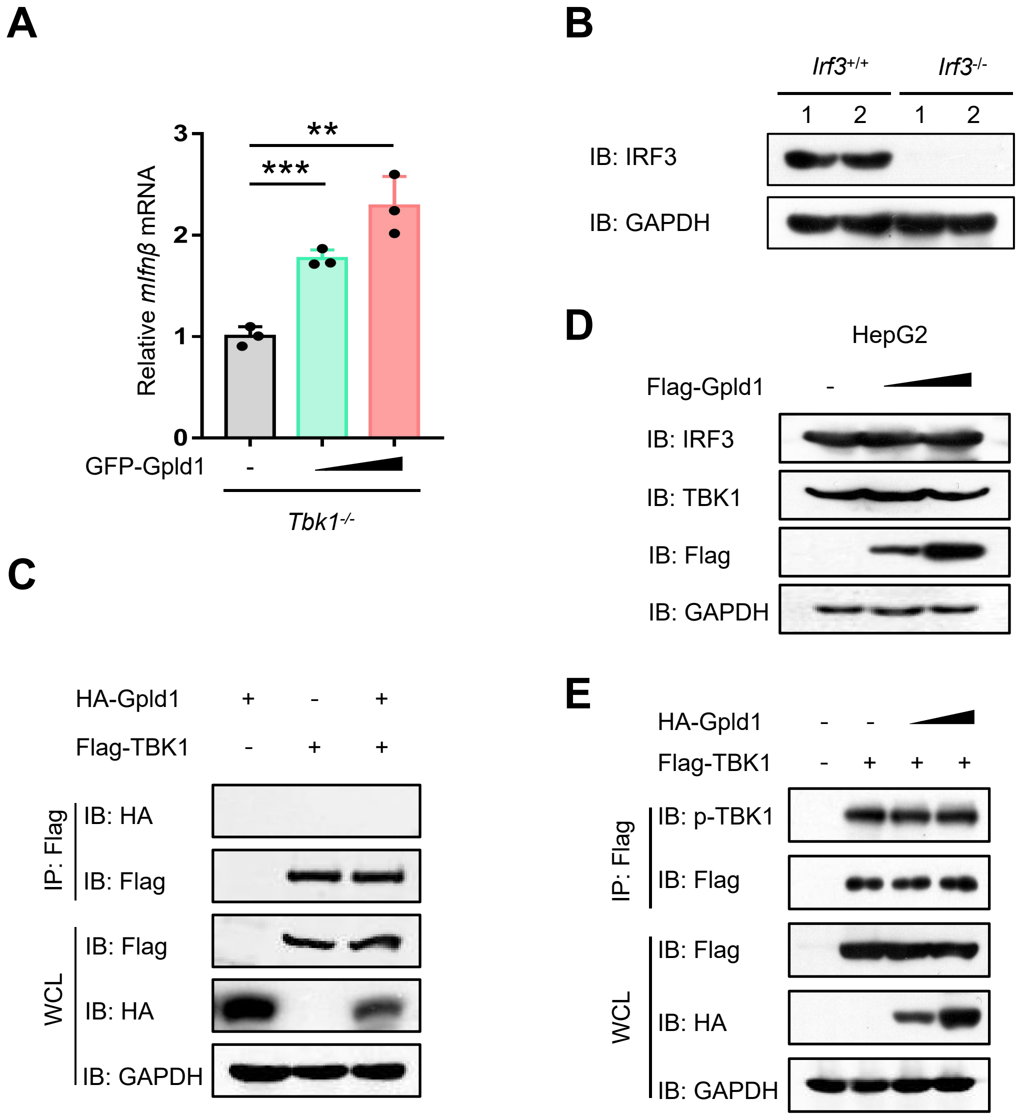
A
B
C
D
E
Relative mIfnβ mRNA
3
2
1
0
***
**
GFP-Gpld1
-
Tbk1-/-
Irf3+/+
Irf3-/-
1
2
1
2
IB: IRF3
IB: GAPDH
HepG2
Flag-Gpld1
-
IB: IRF3
IB: TBK1
IB: Flag
IB: GAPDH
HA-Gpld1
+
-
+
Flag-TBK1
-
+
+
IP: Flag
IB: HA
IB: Flag
WCL
IB: Flag
IB: HA
IB: GAPDH
HA-Gpld1
-
-
Flag-TBK1
-
+
+
+
IP: Flag
IB: p-TBK1
IB: Flag
WCL
IB: Flag
IB: HA
IB: GAPDH

Fig. S3. Gpld1 does not target TBK1.

(A) RT-qPCR analysis of *mIfnβ* mRNA in *Tbk1*$^{-/-}$ MEF cells transfected with the control vector (-) or increasing amounts of GFP-Gpld1.

(B) Western blot analysis of IRF3 protein levels in *Irf3*$^{+/+}$ or *Irf3*$^{-/-}$ HEK293T cells.

(C) Immunoprecipitation analysis of the interaction between Gpld1 and TBK1 in HEK293T cells cotransfected with HA-Gpld1 and Flag-TBK1.

(D) Western blot analysis of TBK1 and IRF3 protein levels in HepG2 cells transfected with the control vector (-) or Flag-Gpld1.

(E) Immunoprecipitation analysis of phosphorylated TBK1 at the Ser172 residue (p-TBK1) in HEK293T cells cotransfected with HA-Gpld1 and Flag-TBK1.

**p<0.01 and ***p<0.001 (two-tailed unpaired Student's t-test). Data are shown as the mean and SD of three biological replicates (A), or are representative of three independent experiments (B-E).

Fig. S4

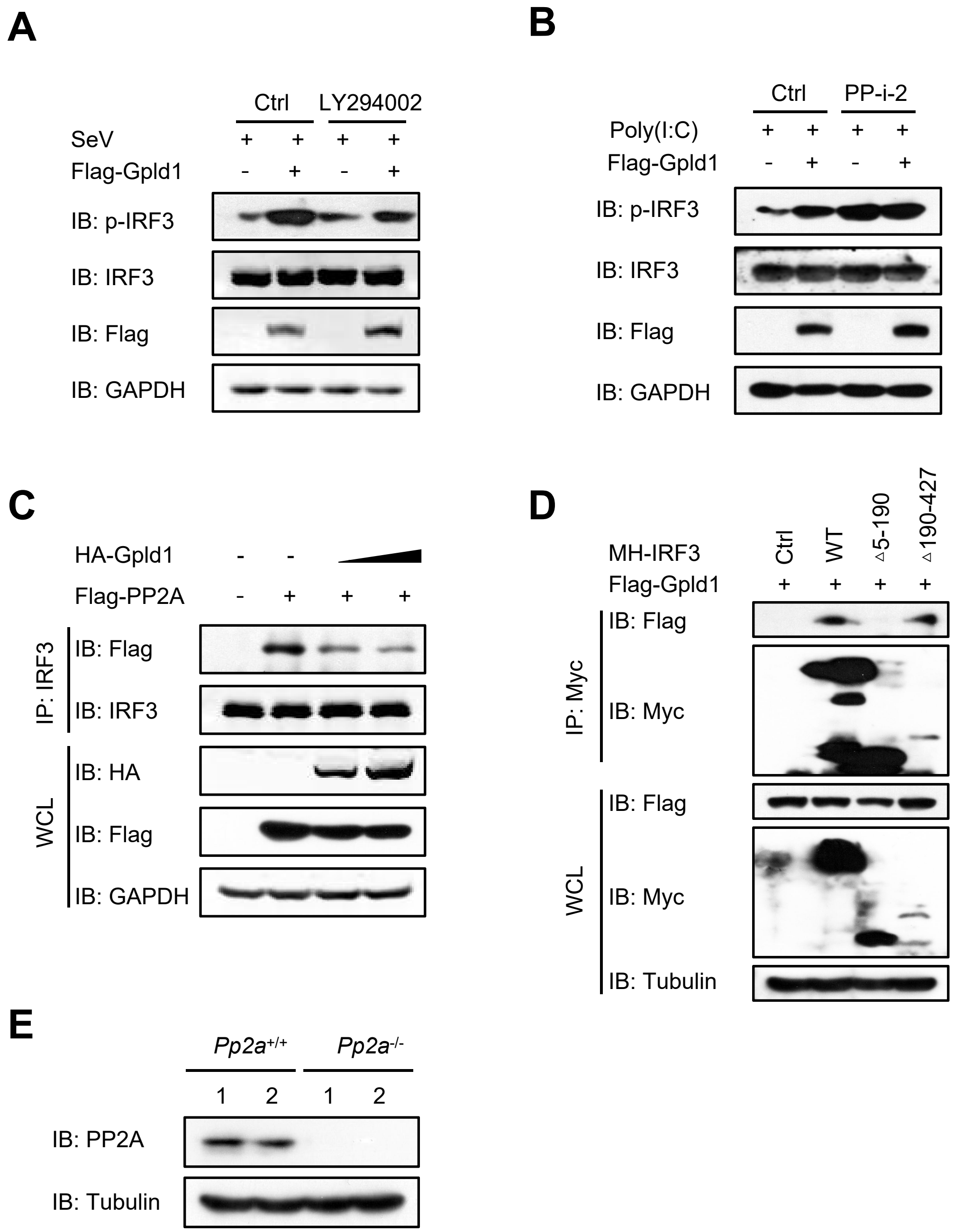
A
Ctrl
LY294002
SeV
Flag-Gpld1
IB: p-IRF3
IB: IRF3
IB: Flag
IB: GAPDH
B
Ctrl
PP-i-2
Poly(I:C)
Flag-Gpld1
IB: p-IRF3
IB: IRF3
IB: Flag
IB: GAPDH
C
HA-Gpld1
Flag-PP2A
IP: IRF3
IB: Flag
IB: IRF3
WCL
IB: HA
IB: Flag
IB: GAPDH
D
MH-IRF3
Ctrl
WT
△5-190
△190-427
Flag-Gpld1
IP: Myc
IB: Flag
IB: Myc
WCL
IB: Flag
IB: Myc
IB: Tubulin
E
Pp2a+/+
Pp2a-/-
1 2 1 2
IB: PP2A
IB: Tubulin

Fig. S4. Gpld1 blocks the binding of PP2A to IRF3.

(A) Western blot analysis of phosphorylated IRF3 at the Ser396 residue (p-IRF3) in 2fTGH cells transfected with Flag-Gpld1 and then treated with LY294002 (5 μM) for 12 h, followed by infection with SeV (MOI=0.1) for 12 h.

(B) Western blot analysis of p-IRF3 in 2fTGH cells transfected with Flag-Gpld1 and then treated with the phosphatase inhibitor cocktail (PP-i-2, Fdbio) for 12 h, followed by transfection with poly(I:C) (1 μg/ml) for 8 h.

(C) Immunoprecipitation analysis of the interaction between IRF3 and PP2A in HEK293T cells cotransfected with HA-Gpld1 and Flag-PP2A.

(D) Immunoprecipitation analysis of the IRF3 domains interacting with Gpld1 in HEK293T cells cotransfected with Flag-Gpld1 and Myc-His (MH)-tagged IRF3 (MH-IRF3; WT or mutants) as indicated.

(E) Western blot analysis of PP2A protein levels in $Pp2a^{+/+}$ or $Pp2a^{-/-}$ HEK293T cells.

Data are representative of three independent experiments (A-E).

Fig. S5

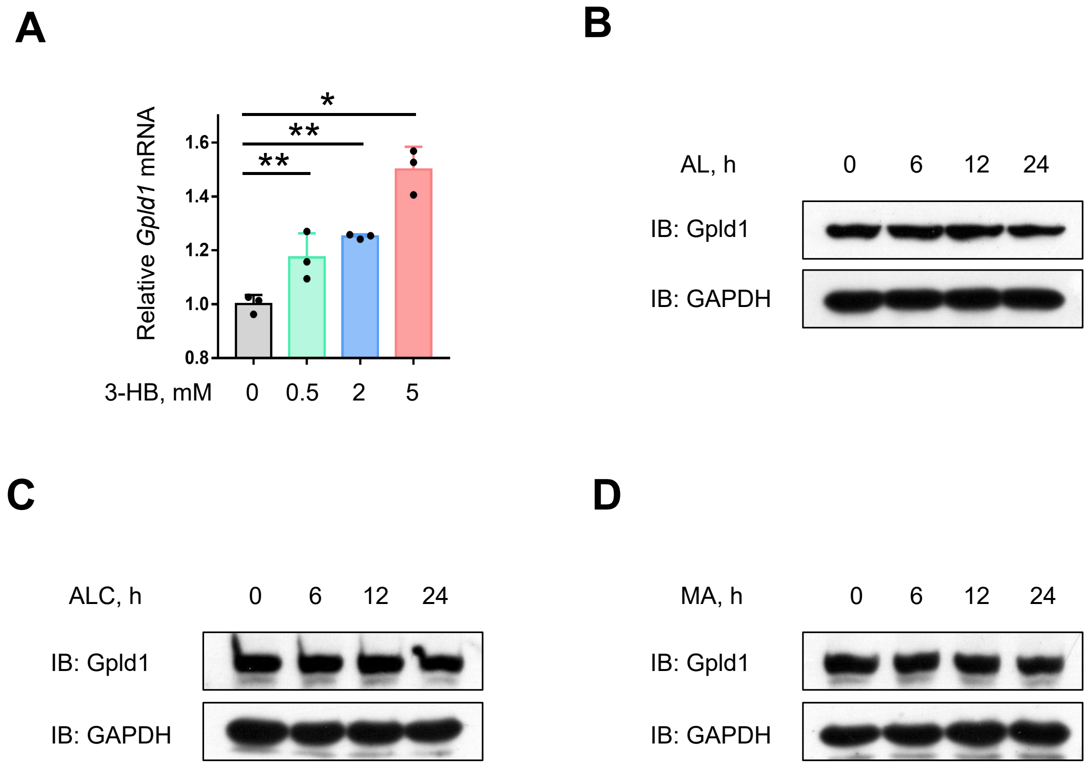
A
B
C
D
Relative Gpld1 mRNA
1.6
1.4
1.2
1.0
0.8
*
**
**
3-HB, mM
0
0.5
2
5
AL, h
0
6
12
24
IB: Gpld1
IB: GAPDH
ALC, h
0
6
12
24
IB: Gpld1
IB: GAPDH
MA, h
0
6
12
24
IB: Gpld1
IB: GAPDH

Fig. S5. The regulation of the protein levels of Gpld1 by the metabolites.

(A) RT-qPCR analysis of *Gpld1* mRNA levels in HepG2 cells treated with 3-HB for 12 h as indicated.

(B) Western blot analysis of Gpld1 protein levels in HepG2 cells treated with AL (2.5 mM) as indicated.

(C) Western blot analysis of Gpld1 protein levels in HepG2 cells treated with ALC (10 μM) as indicated.

(D) Western blot analysis of Gpld1 protein levels in HepG2 cells treated with MA (50 μM) as indicated.

*$p<0.05$ and **$p<0.01$ (two-tailed unpaired Student's t-test). Data are shown as the mean and SD of three biological replicates (A), or are representative of three independent experiments (B-D).

Fig. S6

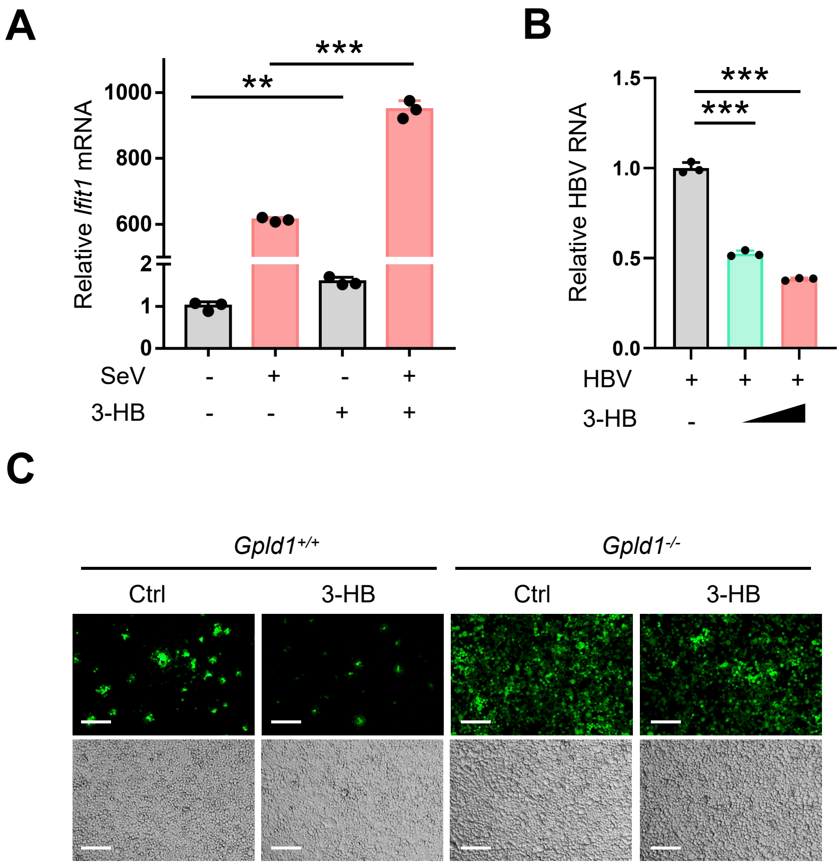
A
Relative Ifit1 mRNA
1000
800
600
2
1
0
**
***
SeV
3-HB
B
Relative HBV RNA
1.5
1.0
0.5
0.0
***
***
HBV
3-HB
C
Gpld1+/+
Gpld1-/-
Ctrl
3-HB
Ctrl
3-HB

Fig. S6. 3-HB regulates IFN-I antiviral immunity via Gpld1.

(A) RT-qPCR analysis of *Ifit1* mRNA levels in HepG2 cells treated with 3-HB (5 mM) for 24 h and then infected with SeV (MOI = 1.0) for 12 h.

(B) RT-qPCR analysis of HBV RNA levels in HepG2 cells transfected with HBV-1.3 constructs and then treated with 3-HB (1 mM or 5 mM) for 24 h.

(C) Fluorescence microscopy of VSV-GFP viruses in *Gpld1*$^{+/+}$ and *Gpld1*$^{-/-}$ HEK293T cells treated with 3-HB (5 mM) for 24 h and then infected with VSV (MOI = 1.0) for 24 h. Scale bars, 200 μm.

$^{**}p<0.01$ and $^{***}p<0.001$ (two-tailed unpaired Student's t-test). Data are shown as the mean and SD of three biological replicates (A, B).

Fig. S7

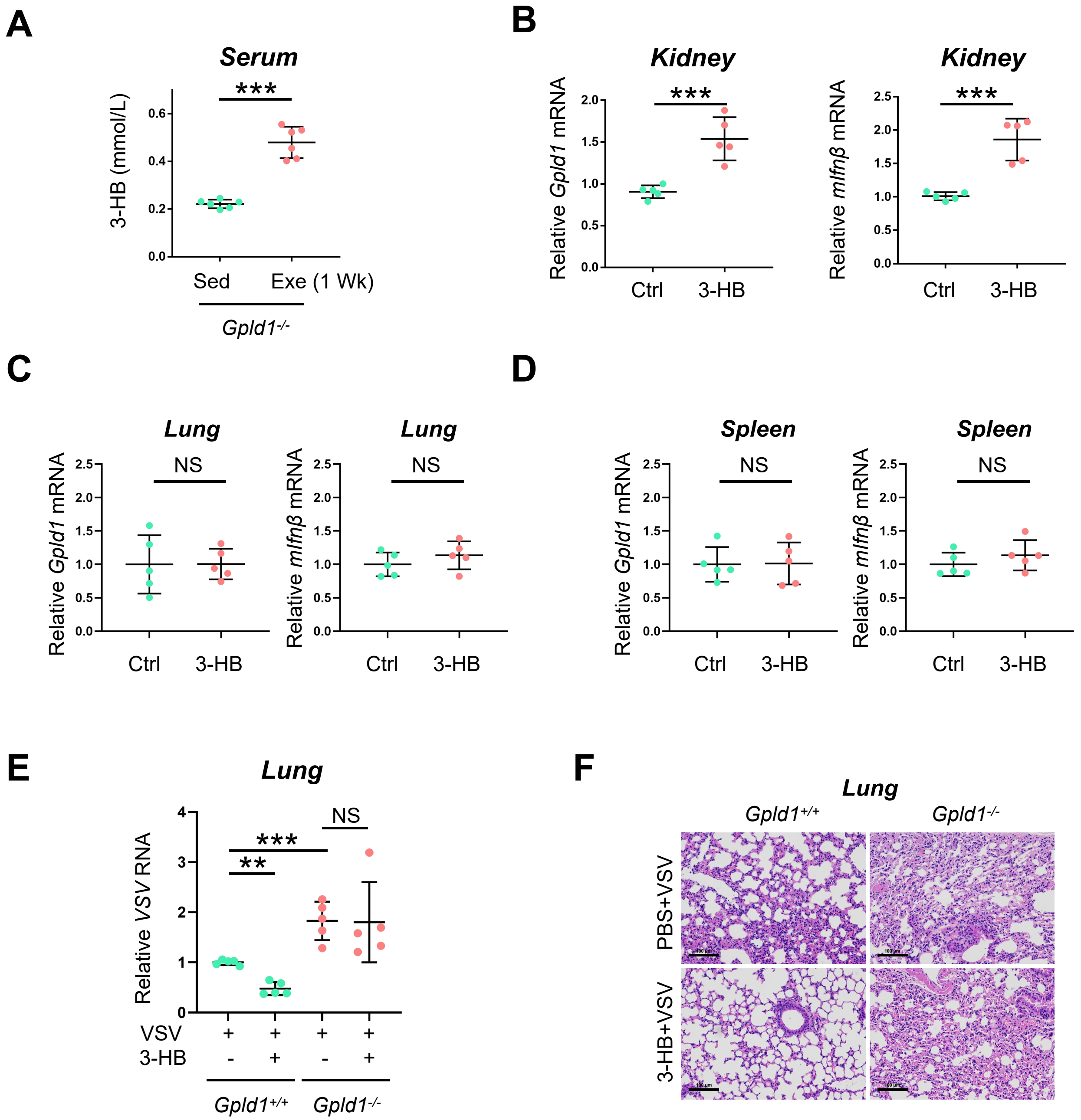

A
Serum
3-HB (mmol/L)
0.0
0.2
0.4
0.6
***
Sed
Exe (1 Wk)
Gpld1-/-
B
Kidney
Relative Gpld1 mRNA
***
Ctrl
3-HB
Kidney
Relative mIfnβ mRNA
***
Ctrl
3-HB
C
Lung
Relative Gpld1 mRNA
NS
Ctrl
3-HB
Lung
Relative mIfnβ mRNA
NS
Ctrl
3-HB
D
Spleen
Relative Gpld1 mRNA
NS
Ctrl
3-HB
Spleen
Relative mIfnβ mRNA
NS
Ctrl
3-HB
E
Lung
Relative VSV RNA
NS
***
**
VSV + + + +
3-HB - + - +
Gpld1+/+
Gpld1-/-
F
Lung
Gpld1+/+
Gpld1-/-
PBS+VSV
3-HB+VSV

Fig. S7. 3-HB promotes the expression of Gpld1 protein and IFN-I *in vivo*.

(A) Colorimetric assay analysis of 3-HB levels in the serum of *Gpld1*$^{-/-}$ mice with one week of exercise (n=6) or sedentary mice (n=6).

(B) RT-qPCR analysis of *Gpld1* and *mIfnβ* mRNA levels in the kidneys of mice administrated with 3-HB (3 mmol/kg, *i.v.*) or PBS (Ctrl).

(C) RT-qPCR analysis of *Gpld1* and *mIfnβ* mRNA levels in the lungs of mice administrated with 3-HB (3 mmol/kg, *i.v.*) or PBS (Ctrl).

(D) RT-qPCR analysis of *Gpld1* and *mIfnβ* mRNA levels in the spleens of mice administrated with 3-HB (3 mmol/kg, *i.v.*) or PBS (Ctrl).

(E) RT-qPCR analysis of VSV RNA levels in the lungs of *Gpld1*$^{+/+}$ and *Gpld1*$^{-/-}$ mice administrated with 3-HB (3 mmol/kg, *i.v.*) or PBS for 24 h, and then infected with VSV (1×10^8 PFU/g of body weight; intranasal) for 48 h.

(F) Hematoxylin and eosin staining of the lungs of *Gpld1*$^{+/+}$ and *Gpld1*$^{-/-}$ mice administrated with 3-HB (3 mmol/kg, *i.v.*) or PBS for 24 h, and then infected with VSV (1×10^8 PFU/g of body weight; intranasal) for 48 h. Scale bars, 100 μm.

NS, not significant ($p>0.05$). $^{**}p < 0.01$, $^{***}p < 0.001$ (two-tailed unpaired Student's t-test). Data are shown as the mean and SD of at least five individual mice (A-E).
